# Approaches to detect genetic effects that differ between two strata in genome-wide meta-analyses: Recommendations based on a systematic evaluation

**DOI:** 10.1371/journal.pone.0181038

**Published:** 2017-07-27

**Authors:** Thomas W. Winkler, Anne E. Justice, L. Adrienne Cupples, Florian Kronenberg, Zoltán Kutalik, Iris M. Heid, the GIANT consortium

**Affiliations:** 1 Department of Genetic Epidemiology, University of Regensburg, Regensburg, Germany; 2 Department of Epidemiology, University of North Carolina, Chapel Hill, NC, United States of America; 3 Department of Biostatistics, Boston University School of Public Health, Boston, MA, United States of America; 4 NHLBI Framingham Heart Study, Framingham, MA, United States of America; 5 Division of Genetic Epidemiology, Department of Medical Genetics, Molecular and Clinical Pharmacology, Medical University of Innsbruck, Innsbruck, Austria; 6 Institute of Social and Preventive Medicine, CHUV-UNIL, Lausanne, Switzerland; 7 Swiss Institute of Bioinformatics, Lausanne, Switzerland; McMaster University, CANADA

## Abstract

Genome-wide association meta-analyses (GWAMAs) conducted separately by two strata have identified differences in genetic effects between strata, such as sex-differences for body fat distribution. However, there are several approaches to identify such differences and an uncertainty which approach to use. Assuming the availability of stratified GWAMA results, we compare various approaches to identify between-strata differences in genetic effects. We evaluate type I error and power via simulations and analytical comparisons for different scenarios of strata designs and for different types of between-strata differences. For strata of equal size, we find that the genome-wide test for difference without any filtering is the best approach to detect stratum-specific genetic effects with opposite directions, while filtering for overall association followed by the difference test is best to identify effects that are predominant in one stratum. When there is no a priori hypothesis on the type of difference, a combination of both approaches can be recommended. Some approaches violate type I error control when conducted in the same data set. For strata of unequal size, the best approach depends on whether the genetic effect is predominant in the larger or in the smaller stratum. Based on real data from GIANT (>175 000 individuals), we exemplify the impact of the approaches on the detection of sex-differences for body fat distribution (identifying up to 10 loci). Our recommendations provide tangible guidelines for future GWAMAs that aim at identifying between-strata differences. A better understanding of such effects will help pinpoint the underlying mechanisms.

## Introduction

Genome-wide association studies (GWAS) and genome-wide association meta-analyses (GWAMAs) are one of the most successful approaches to identify genetic regions that are relevant for complex phenotypes and diseases [1]. Usually in GWAMAs, a group responsible for meta-analyses develops an analysis plan describing each of the models to be conducted by participating studies and distributes it to study analysts; the study analysts then conduct the specified study-specific GWAS and the meta-analysts collect, quality control, and meta-analyze the study-specific aggregated statistics across studies [2]. To not burden study partners too much, the study-specific analysis models are generally sparse, clearly described, and easy to conduct with available software in a standardized way.

Recently, the identification of genetic loci where the genetic effects are modulated by non-genetic factors such as sex or life-style factors (*gene-environment interaction*, GxE), became a major focus [3]. When the “environmental” factor is dichotomous—such as sex or ever versus never smoking—the interaction effect is equivalent to a difference of the genetic effect between the two groups (*genetic effect with between-strata difference*, GxS). Many GWAMA consortia do not search genome-wide for variants with GxS, but restrict their test for GxS on genetic variants that are identified with an overall genetic effect on the phenotype of interest [4–7]. However, this approach would have little chance to detect an effect in opposite directions for the two strata. Only few consortia conduct GWAMAS based on models including an interaction effect [8] (Rao et al. 2017, accepted at Circulation Cardiovascular genetics). Notably, such interaction models can become particularly complex when further covariates are involved [9]. While such interaction models are theoretically feasible, they are also logistically challenging as the more complex models and limitations of GWAS software to extract multiple covariate estimates hamper the study analysts to conduct the analyses smoothly and correctly.

Several consortia conduct *stratified GWAMAS*, where study analysts are asked to perform the analyses separately by stratum—for example separately for men and women or for persons with and without diabetes [4,5,7,10]. For study analysts, this is relatively straight forward to implement with existing genome-wide analysis software. For meta-analysts, stratified GWAS allow a stratified meta-analyses, opening up multiple options: (i) to test for stratum-specific effects *(stratified association test)*, (ii) to combine stratified results together and to test for stratum-combined effects (*overall association test* [11]), (iii) to test for difference between stratum-specific effects (*difference test* [12]), or (iv) to test for joint effects accounting for potential GxS by using the sum of squared stratum-specific test statistics (*alternative joint test* [13]). The *alternative joint test* was shown to be equivalent to the *joint test* combining the main and the interaction effect for a dichotomous factor S [8]. Numerous variants with GxS have already been identified via stratified GWAMAs [8,14,15], applying different approaches to search for GxS. While the difference test is always the ultimate test to establish GxS and frequently utilized to search for GxS [12], there is also previous work where genetic variants are filtered prior to the difference testing using the overall [7,10], the stratified [12], or the joint test as a filter [8], allegedly to increase power. Still, the power to detect GxS depends on the type of GxS interaction: whether the effects point into opposite directions in the two strata (qualitative), the effect is zero in one stratum and significant in the other (pure), or the effects are directionally consistent being larger in one stratum (quantitative). A systematic evaluation of all approaches and a recommendation as to which approach should be utilized—with or without making assumptions on the type of GxS—are lacking.

Here, we conduct a systematic comparison of approaches to identify GxS based on stratified GWAMAs for a continuous outcome with regard to type I error and power. We exemplify the impact of the different approaches on the identification of sexually dimorphic variants for body fat distribution using real data from the GIANT consortium [12].

## Materials and methods

### Notation and models

We consider K studies and 2 strata, with a total sample size of *n = n*_*1*_*+n*_*2*_, *n*_*i*_ = sum_k_(*n*_*ik*_), *k* = 1…,*K*, *i* = 1,2 strata, and *f = n*_*2*_*/n*_*1*_. For an individual *j*, $Y_{ik}^{\left(j\right)}$ denotes a continuous phenotype value and $G_{ik}^{\left(j\right)}$ = 0,1,2 the number of alleles for a genetic variant (omitting the indexing of the millions of variants analyzed). A stratified GWAMA involves two steps, the study- and stratum-specific GWAS conducted by the study analyst and the stratum-specific meta-analysis.

For *stratified GWAS*, the linear regression model computed per stratum for each of the genetic variants (omitting further covariates) can be written as

$$Y_{ik}^{\left(j\right)}=\propto_{ik}+\beta_{ik}G_{ik}^{\left(j\right)}+E_{ik}^{\left(j\right)},$$ (1)

with *α*_*ik*_ denoting the intercept, *β*_*ik*_ the genetic effect and $E_{ik}^{\left(j\right)}∼N(0,\sigma_{Eik}^{2})$. We assume that phenotypes have been normalized to have zero mean and unit variance in each study and stratum (i.e., $\sigma_{Yik}^{2}=\sigma_{Y}^{2}=1$). We also assume similar minor allele frequencies across studies and strata (*MAF*_*ik*_ *≈ MAF*) and thus similar genotype variances, $\sigma_{Gik}^{2}=\sigma_{G}^{2}$ = 2 * *MAF* * (1-*MAF*), i.e. that the SNP is not associated with the stratum variable and homogeneous across studies (**S1 Note**). Our notation here assumes that the studies include only unrelated individuals (see below for the extension to related individuals).

For the stratum-specific meta-analysis per variant, pooled genetic effect estimates, ${\hat{\beta }}_{i}$, and standard errors, *se*_*i*_, are computed via an inverse-variance weighted meta-analysis by stratum [16], assuming equal genetic effects across studies, since we focus on identification rather than quantification of genetic effects [17]. Under the assumption that the studies are from similar populations with similar genotypic variance, the inverse-variance weighted meta-analyzed ${\hat{\beta }}_{i}$ and *se*_*i*_ are approximately identical to an estimate derived from one single large mega-study [18,19].

### Stratified GWAMA approaches to identify G x S

Our stratified GWAMA approaches are based on four statistical tests. The statistical test to identify GxS is the *difference test*, $Z_{Diff}=\left({\hat{\beta }}_{1}-{\hat{\beta }}_{2}\right)/\sqrt{{se}_{1}^{2}+{se}_{2}^{2}}$. This is under the assumption of no relatedness of subjects across strata and thus no correlation of ${\hat{\beta }}_{1}$ with ${\hat{\beta }}_{2}$. Under the assumption of unrelated individuals across strata and no latent covariate interacting with a dichotomous factor S, the difference test is equivalent to testing interaction of the genetic effect with a dichotomous factor S. We consider three further tests that are utilized to filter genetic variants prior to the difference testing: filtering on (i) *overall association*, $Z_{Overall}=\left({\hat{\beta }}_{1}/{se}_{1}^{2}+{\hat{\beta }}_{2}/{se}_{2}^{2}\right)/\sqrt{1/{se}_{1}^{2}+1/{se}_{2}^{2}}$, (ii) *stratified association*, $Z_{1}={\hat{\beta }}_{1}/{se}_{1}$ or $Z_{2}={\hat{\beta }}_{2}/{se}_{2}$, or (iii) the *alternative joint association*, $C_{Joint}={\left({\hat{\beta }}_{1}/{se}_{1}\right)}^{2}+{\left({\hat{\beta }}_{2}/{se}_{2}\right)}^{2}$. All test statistics can be computed based on stratified GWAMA results, i.e. stratum-specific pooled genetic effect, ${\hat{\beta }}_{i}$, and corresponding standard errors, *se*_*i*_, i = 1,2 (see **S1 Table** for a detailed description of the four tests).

The tests are used to generate various approaches to identify GxS. In the approach without filtering, the difference test is applied genome-wide, $\left[{\mathrm{Diff}}_{\alpha_{\mathrm{Diff}}}\right]$, and GxS can readily be identified using a genome-wide significance level of *α*_*Diff*_ = 5x10^-8^ (= 0.05/1,000,000) [20]. In the approaches with filtering, one of the three filtering tests is applied genome-wide, variants with a p-value below a *filtering threshold*, *α*_*Filter*_, are selected, and lead variants are extracted (variant with lowest P-value within +/- 500,000 base positions). When *M* lead variants are selected by the filtering, these variants have an established association with the phenotype, but GxS has yet to be ascertained by the difference test using a Bonferroni-corrected significance level, *α*_*Diff*_ = 0.05/*M* (**S1 Methods**). The stricter the filtering threshold, the smaller *M*, thus decreasing the multiple testing burden; however, a stricter filtering threshold might miss true GxS signals. We will thus consider varying filtering thresholds.

When overall, stratified or joint association filtering is applied to the same stratified GWAMA results as the difference test (*one-stage approach*), the approaches to detect GxS are denoted here as $\left[{\mathrm{Overall}}_{\alpha_{\mathrm{Filter}}}\to {\mathrm{Diff}}_{\alpha_{\mathrm{Diff}}}\right]$, $\left[{\mathrm{Strat}}_{\alpha_{\mathrm{Filter}}}\to {\mathrm{Diff}}_{\alpha_{\mathrm{Diff}}}\right]$ or $\left[{\mathrm{Joint}}_{\alpha_{\mathrm{Filter}}}\to {\mathrm{Diff}}_{\alpha_{\mathrm{Diff}}}\right]$, respectively. When the filtering test is statistically dependent on the difference test the tests have to be applied to two independent sets of stratified GWAMA results (*two-stage approach*) to achieve appropriate type 1 error control. To obtain two independent stratified GWAMA results, the available GWAS studies are to be separated into a first and a second stage and meta-analyzed by stage. Then the filtering is to be conducted in the first stage meta-analysis and the difference test in the second stage meta-analysis. We denote the respective two-stage approaches to detect GxS as $\left[{\mathrm{Overall}}_{\alpha_{\mathrm{Filter}}}]\to [{\mathrm{Diff}}_{\alpha_{\mathrm{Diff}}}\right]$, $\left[{\mathrm{Strat}}_{\alpha_{\mathrm{Filter}}}]\to [{\mathrm{Diff}}_{\alpha_{\mathrm{Diff}}}\right]$ or $\left[{\mathrm{Joint}}_{\alpha_{\mathrm{Filter}}}]\to [{\mathrm{Diff}}_{\alpha_{\mathrm{Diff}}}\right]$.

We explore the three filtering tests followed by the difference test both as one-stage and as two-stage approaches. Together with the difference test without filtering, this yields a total of seven approaches to detect GxS in our systematic evaluation (an overview of approaches is shown in **S1 Fig**).

### Extending to related individuals

When a study includes related individuals, this can be accounted for within each stratified GWAS model and thus within each stratum by extending to mixed models [21]. Relatedness across studies within the same stratum can be handled via generalized meta-analysis [22]. Including related individuals across strata yields correlated stratum-specific (variant-specific) estimates (${\hat{\beta }}_{1}$ and ${\hat{\beta }}_{2}$). This correlation can be estimated by the Pearson correlation coefficient of all ${\hat{\beta }}_{1}$ and ${\hat{\beta }}_{2}$ estimates across all genetic variants, denoted by *r*.

The test statistics for the difference test can then be extended to $Z_{Diff}=\left({\hat{\beta }}_{1}-{\hat{\beta }}_{2}\right)/\sqrt{{se}_{1}^{2}+{se}_{2}^{2}-2∙Cov({\hat{\beta }}_{1},{\hat{\beta }}_{2})}$ and the $Cov({\hat{\beta }}_{1},{\hat{\beta }}_{2})$ can be estimated by *r* ∙ *se*_1_ ∙ *se*_2_. In the case of related individuals (r > 0), this correction yields more extreme test statistics compared to when relatedness is ignored and will thus have better power to detect a difference. When the individuals are unrelated (r close to 0), this formula is the same as the one given in the previous chapter. There is no disadvantage of using this extended formula in all circumstances.

For the filtering with the overall or the joint test, the inclusion of related subjects between strata without accounting for them will yield underestimated standard errors and therefore deflated p-values and larger type I error. Thus, when filtering at a threshold at, e.g. 5x10^-5^, the filtering will be less stringent allowing for more variants to pass. The filtering by the stratified test is unaffected by the inclusion of such related subjects.

### Simulation-based evaluation of type I error

Based on simulated data, we estimate the type I error rate of each of the seven stratified GWAMA approaches to detect GxS. We simulate one large data set of 200,000 unrelated individuals under the null hypothesis of no GxS i.e. no difference between stratum-specific effects (*H*_0_: *β*_1_ = *β*_2_ = *β*). We implement several scenarios of varying values for *β*, varying *MAF* (0.05, 0.30) and varying strata designs (balanced design, *f* = 1; unbalanced designs *f* = 1/3, 3) (details in **S2 Methods)**.

For each scenario and approach, we estimate the type I error rate for a 5% significance level as the proportion of nominally significant variants (*P*_*Diff*_ < 0.05) relative to the number of conducted difference tests (# significant variants/1,000,000 without filtering, # significant variants/*M* with filtering).

### Analytical computation of power

For all stratified GWAMA approaches to detect GxS that have valid type I error, we compute power analytically. As a measure of the stratum-specific genetic effect, we introduce

$$R_{i}=b_{i}\frac{\sigma_{G}}{\sigma_{Y}}$$ (2)

For each stratum, $R_{i}^{2}$ represents the phenotypic variance explained by the variant; *R*_*i*_ denotes the direction of the effect as does *b*_*i*_, which can be opposite in the two strata (*qualitative GxS*), zero in one stratum and significant in the other (*pure GxS*), or directionally consistent with different quantity (*quantitative GxS*). Analytical power formulae are provided in the **S3 Methods** for the difference test, *Power*_*Diff*_, and for each of the filtering tests, *Power*_*Filter*_, for varying *n*_*1*_, *f*, *R*_*1*_, *R*_*2*_, and *α*, where *n*_*1*_ reflects the sample size of the stratum with the larger absolute effect (|*R*_*2*_| < = |*R*_*1*_|); *α* is the filtering threshold, *α*_*Filter*_, for the filtering tests or the significance level, *α*_*Diff*_, for the difference test. When the filtering test and difference test are independent or when they are applied to two different stages of meta-analysis results, the power of the approach can be derived as the product of the power of the filtering test and the power of the difference test, i.e. *Power*_*Approach*_ = *Power*_*Filter*_ ∙ *Power*_*Diff*_ (**S3 Methods**). Of note, *n*_*1*_ and *n*_*2*_ reflect the stratum-specific effective number of subjects for related subjects can be computed by the method proposed for correlated SNPs [23].

We compute the power of each approach for various realistic scenarios varying strata design and varying genetic effect sizes, motivated by the GIANT data with *n* = 200,000. We also varied the types of GxS (qualitative, pure, quantitative) and the filtering threshold (details in **S4 Methods**).

### Genetic Investigation of Anthropometric Traits (GIANT) consortium

To exemplify the impact of the different stratified GWAMA approaches to detect GxS, we utilize the sex-stratified GWAMA results for WHRadjBMI from GIANT [12]. This data comprises up to 77,000 men and 98,000 women from two independent stages that derive from the fact that the GWAS data was collected in two waves. The GWAMA results contain pooled sex-specific genetic effect estimates and standard errors for ~2.8 million variants. We apply each of the stratified GWAMA approaches with valid type I error to detect variants with between-sex difference, using the two stages for the two-stage approaches and meta-analyzing the stage-specific estimates by sex to generate one set of meta-analyzed results for each sex for the one-stage approaches. Given that the majority of the 2.8 million variants can be considered as not associated, we evaluate the empirical type I error by QQ plots and by calculating genomic control inflation factors (λ_GC_) [24]. We also derive the number of sexually dimorphic loci for WHRadjBMI based on each of the approaches, to demonstrate the approaches taken.

## Results

### Overview

**Fig 1:**
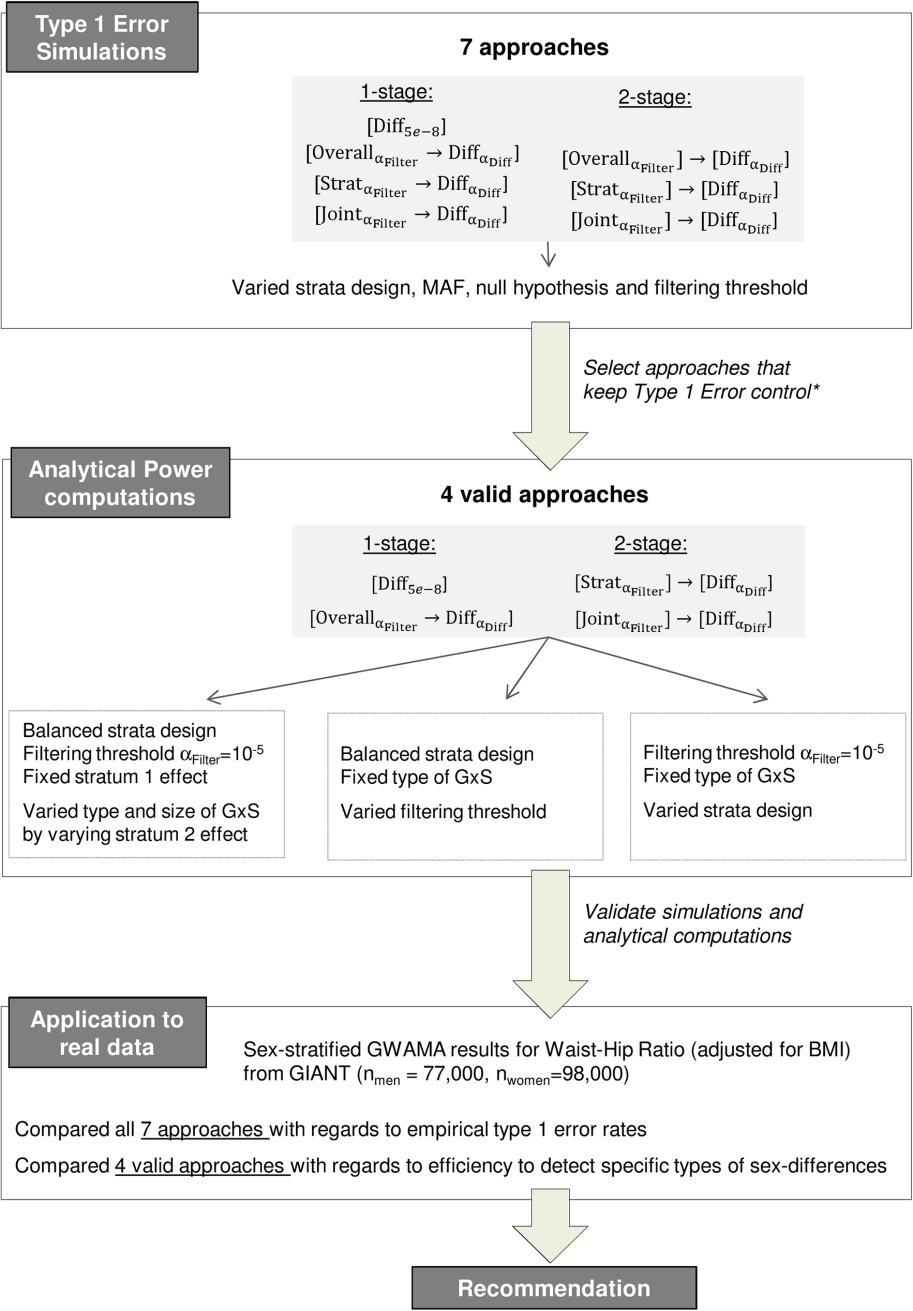
Workflow of the conducted analyses. The figure shows an overview on the four major project steps that were outlined subsequently: 1) Simulations to evaluate Type 1 Error, 2) Analytical computations of power, 3) Real data application and 4) Recommendation. *The valid 2-stage approach $\left[{\mathrm{Overall}}_{\alpha_{Filter}}]\to [{\mathrm{Diff}}_{\alpha_{\mathrm{Diff}}}\right]$ was omitted from power computations due to expectedly lower power of the approach compared to the valid 1-stage approach $\left[{\mathrm{Overall}}_{\alpha_{Filter}}\to {\mathrm{Diff}}_{\alpha_{\mathrm{Diff}}}\right]$ that makes use of the full available sample size for both filtering and difference testing.

We examine type I error and power of stratified GWAMA approaches to detect GxS. We aim at identifying the best approach, which is the approach that maintains type I error and exhibits the best power, for various strata designs (balanced, unbalanced) with and without an a priori hypothesis of a given type of GxS (qualitative, pure, quantitative). We exemplify the impact of each approach on the identification of sexually dimorphic genetic variants for WHRadjBMI based on the sex-stratified GWAMA results from GIANT. A summary of the workflow is shown in **Fig 1**.

### Type I error of the stratified GWAMA approaches to detect GxS

In order to derive the empirical type I error for the seven stratified GWAMA approaches to detect GxS, we simulate genetic association data first under a balanced strata design with *n*_*1*_ = 100,000, *n*_*2*_ = 100,000. Our results show that the difference test without filtering, $\left[{\mathrm{Diff}}_{\alpha_{\mathrm{Diff}}}\right]$, when judged at a significance level of 0.05, keeps type I error control (4.97% to 5.02%, **Table 1**). Among the three one-stage approaches with filtering, we yield a valid type I error for the overall association filtering, $\left[{\mathrm{Overall}}_{\alpha_{\mathrm{Filter}}}\to {\mathrm{Diff}}_{\alpha_{\mathrm{Diff}}}\right]$, but a severe violation of type I error control for the stratified test filtering and the alternative joint test filtering, $\left[{\mathrm{Strat}}_{\alpha_{\mathrm{Filter}}}\to {\mathrm{Diff}}_{\alpha_{\mathrm{Diff}}}\right]$ and $\left[{\mathrm{Joint}}_{\alpha_{\mathrm{Filter}}}\to {\mathrm{Diff}}_{\alpha_{\mathrm{Diff}}}\right]$ (type I error from 8.83% to 49.9%). When applied in two stages, all three filtering approaches, $\left[{Overall}_{\alpha_{Filter}}]\to {[Diff}_{\alpha_{Diff}}\right]$, $\left[{\mathrm{Strat}}_{\alpha_{\mathrm{Filter}}}]\to {[\mathrm{Diff}}_{\alpha_{\mathrm{Diff}}}\right]$, or $\left[{\mathrm{Joint}}_{\alpha_{\mathrm{Filter}}}]\to {[\mathrm{Diff}}_{\alpha_{\mathrm{Diff}}}\right]$, keep type I error control as expected (type I error from 4.90% to 5.18%, **Table 1**). These results are supported by QQ-plots depicting the observed distribution of the difference test P-values versus the expected, which show that the number of observed P-values matches the expected very well across the full range of values (**S2 Fig and S3 Fig**). For unbalanced strata designs, we observe similar results (**S4 Fig and S5 Fig**). Our results demonstrate that the stratified and the alternative joint tests are statistically not independent of the difference test, while the overall association is independent.

**Table 1:** Simulation-based Type I error for the seven stratified GWAMA approaches to detect GxS. Shown is the type I error at a 5% significance level derived from simulated data as the proportion of variants with nominally significant difference test (*P*_*Diff*_<0.05) relative to the number of variants tested for difference (1,000,000 in the difference test without filtering, number of filtered variants in the approaches with filtering). The simulation results are based on a balanced strata design (*n*_*1*_ = 100,000, *n*_*2*_ = 100,000; split in half for two-stage approaches), variants with *MAF* = 0.05 or 0.30, and phenotypes simulated under the null hypothesis of no GxS, i.e. no difference between stratum-specific effects (*H*_0_: *β*_1_ = *β*_2_ = *β*). We present the results for *β* = 0 and *β* ≠ 0. For the second setting, we set *β* as the minimum effect size detectable at 80% power for the given *MAF* and the given sample size for the difference test (*n* = 200 000 for one-stage approaches, β = 0.029, 0.014 for *MAF* = 0.05, *MAF* = 0.30, respectively; *n*_*Stage*_ = 100,000 for the two-stage approaches, β = 0.041, 0.019 for *MAF* = 0.05, *MAF* = 0.30, respectively). Marked in bold are violated type 1 error rates.

		*β* = 0			*β* ≠ 0		
Approach	MAF	#variants in difference test^a^	#variants with P_Diff_<0.05^b^	Type I Error [%]	#variants in difference test^a^	#variants with P_Diff_<0.05^b^	Type I error [%]
*Approach without filtering*							
$\left[{\mathrm{Diff}}_{\alpha_{\mathrm{Diff}}}\right]$	0.05	1 000 000	49 882	4.99	1 000 000	49 652	4.97
	0.30	1 000 000	49 949	4.99	1 000 000	50 207	5.02
*One-stage filtering approaches*							
$\left[{\mathrm{Overall}}_{\alpha_{\mathrm{Filter}}}\to {\mathrm{Diff}}_{\alpha_{\mathrm{Diff}}}\right]$	0.05	50 032	2 454	4.90	323 857	16 143	4.98
	0.30	49 879	2 497	5.01	324 431	16 323	5.03
$\left[{\mathrm{Strat}}_{\alpha_{\mathrm{Filter}}}\to {\mathrm{Diff}}_{\alpha_{\mathrm{Diff}}}\right]$	0.05	49 018	20 956	**42.8**	76 496	21 732	**28.4**
	0.30	49 057	20 912	**42.6**	76 415	22 094	**28.9**
$\left[{\mathrm{Joint}}_{\alpha_{\mathrm{Filter}}}\to {\mathrm{Diff}}_{\alpha_{\mathrm{Diff}}}\right]$	0.05	49 809	24 732	**49.7**	235 152	20 762	**8.83**
	0.30	49 667	24 784	**49.9**	235 383	21 076	**8.95**
*Two-stage filtering approaches*							
$\left[{\mathrm{Overall}}_{\alpha_{\mathrm{Filter}}}]\to [{\mathrm{Diff}}_{\alpha_{\mathrm{Diff}}}\right]$	0.05	49 812	2 475	4.97	16 346	801	4.90
	0.30	49 726	2 548	5.12	16 291	801	4.92
$\left[{\mathrm{Strat}}_{\alpha_{\mathrm{Filter}}}]\to [{\mathrm{Diff}}_{\alpha_{\mathrm{Diff}}}\right]$	0.05	49 249	2 475	5.03	3 780	189	5.00
	0.30	49 306	2 459	4.99	3 786	196	5.18
$\left[{\mathrm{Joint}}_{\alpha_{\mathrm{Filter}}}]\to {[\mathrm{Diff}}_{\alpha_{\mathrm{Diff}}}\right]$	0.05	49 948	2 470	4.95	11 812	562	4.76
	0.30	49 976	2 504	5.01	11 749	601	5.12

^a^ Number of independent variants tested for difference.

^b^ Number of variants with nominally significant difference (*P*_*Diff*_ < 0.05); *MAF* = minor allele frequency.

In summary, the difference test without filtering and the overall association filtering prior to the difference testing are the only valid one-stage approaches, and all two-stage approaches are valid, yielding five valid approaches altogether: $\left[{\mathrm{Diff}}_{\alpha_{\mathrm{Diff}}}\right]$, $\left[{\mathrm{Overall}}_{\alpha_{\mathrm{Filter}}}\to {\mathrm{Diff}}_{\alpha_{\mathrm{Diff}}}\right]$, $\left[{Overall}_{\alpha_{Filter}}]\to {[Diff}_{\alpha_{Diff}}\right]$, $\left[{\mathrm{Strat}}_{\alpha_{\mathrm{Filter}}}]\to {[\mathrm{Diff}}_{\alpha_{\mathrm{Diff}}}\right]$ or $\left[{\mathrm{Joint}}_{\alpha_{\mathrm{Filter}}}]\to {[\mathrm{Diff}}_{\alpha_{\mathrm{Diff}}}\right]$. Since the one-stage approach $\left[{\mathrm{Overall}}_{\alpha_{\mathrm{Filter}}}\to {\mathrm{Diff}}_{\alpha_{\mathrm{Diff}}}\right]$ is valid and the two-stage approach $\left[{Overall}_{\alpha_{Filter}}]\to {[Diff}_{\alpha_{Diff}}\right]$ is not expected to be more powerful, we will ignore the latter and focus in the following on the remaining four valid approaches.

### Power of stratified GWAMA approaches to detect GxS under a balanced strata design

Next, we compare the power of the selected four approaches to detect GxS, $\left[{\mathrm{Diff}}_{\alpha_{\mathrm{Diff}}}\right]$, $\left[{\mathrm{Overall}}_{\alpha_{\mathrm{Filter}}}\to {\mathrm{Diff}}_{\alpha_{\mathrm{Diff}}}\right]$, $\left[{\mathrm{Strat}}_{\alpha_{\mathrm{Filter}}}]\to {[\mathrm{Diff}}_{\alpha_{\mathrm{Diff}}}\right]$ and $\left[{\mathrm{Joint}}_{\alpha_{\mathrm{Filter}}}]\to {[\mathrm{Diff}}_{\alpha_{\mathrm{Diff}}}\right]$, for a balanced strata design (*n*_*1*_ = 100,000, *n*_*2*_ = 100,000) for varying stratum-specific effect sizes, R_1_ and R_2_, and varying types and sizes of GxS. We assume a genome-wide significance level for the difference test without filtering (*α*_*Diff*_ = 5 x 10^−8^) and, for the approaches with filtering, a filtering threshold of *α*_*Filter*_ = 1 x 10^−5^ with a Bonferroni-corrected significance level at 0.05/*M* for the subsequent difference test (*M* being the number of filtered variants).

**Fig 2:**
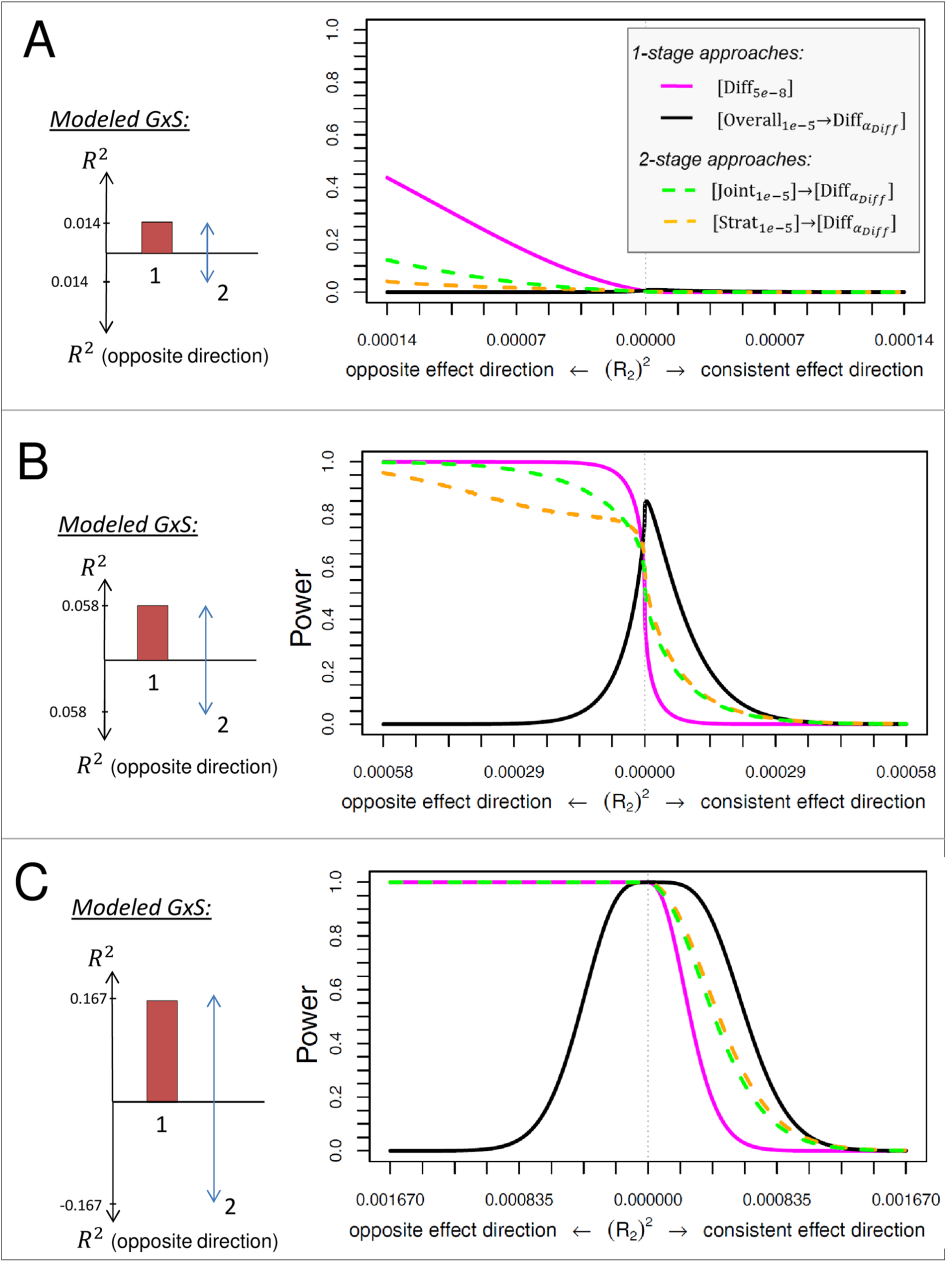
Power of stratified GWAMA approaches to identify GxS for balanced strata design. Shown is the power to detect GxS in equally sized strata (*n*_*1*_ = 100,000, *n*_*2*_ = 100,000) for each of the considered approaches, for varying effect sizes in stratum 2, $R_{2}^{2}$, with a fixed genetic effect in stratum 1, $R_{1}^{2}$, that is (A) small ($R_{1}^{2}=0.014\%$), (B) medium ($R_{1}^{2}=0.058\%$), or (C) large ($R_{1}^{2}=0.167\%$). The effect sizes for $R_{1}^{2}$ are chosen as those observed for WHRadjBMI near *STAB1*, *PPARG* or *LYPLAL1*, respectively. The modeled GxS are visualized on the left side (red bar: $R_{1}^{2}$, blue arrows: varying $R_{2}^{2}$). For the difference test without filtering, we assume a significance level at 5 x 10^−8^; for approaches with filtering, the filtering threshold is 1 x 10^−5^ and the significance level applied for the consecutive difference test is *α*_*Diff*_ = *M*/0.05, with M being the number of filtered lead variants (see Methods).

Our computations show that the power of an approach largely depends on the type of GxS (**Fig 2A–2C**): when the effects point into opposite direction (qualitative GxS), the difference test, [Diff_5e-8_], and the two-stage approaches $\left[{\mathrm{Strat}}_{1\mathrm{e-}5}]\to [{\mathrm{Diff}}_{\alpha_{\mathrm{Diff}}}\right]$ and $\left[{\mathrm{Joint}}_{1\mathrm{e-}5}]\to [{\mathrm{Diff}}_{\alpha_{\mathrm{Diff}}}\right]$ perform substantially better than the overall association filtering approach, $\left[{\mathrm{Overall}}_{1\mathrm{e-}5}\to {\mathrm{Diff}}_{\alpha_{\mathrm{Diff}}}\right]$. In contrast, when the effects point into the same direction (quantitative GxS) or are only pronounced in one stratum (pure GxS), the overall association filtering approach shows much better power than the other three approaches. For the example of the medium sized *R*_*1*_ (**Fig 2B**), the power to detect a pure GxS is 80.8% for $\left[{\mathrm{Overall}}_{1\mathrm{e-}5}\to {\mathrm{Diff}}_{\alpha_{\mathrm{Diff}}}\right]$, and only 47.4%, 61.1%, or 55.2%, for [Diff_5e-8_], $\left[{\mathrm{Strat}}_{1\mathrm{e-}5}]\to [{\mathrm{Diff}}_{\alpha_{\mathrm{Diff}}}\right]$ or $\left[{\mathrm{Joint}}_{1\mathrm{e-}5}]\to [{\mathrm{Diff}}_{\alpha_{\mathrm{Diff}}}\right]$, respectively. When the predominant effect (stratum 1) is large enough (**Fig 2C**), a quantitative or pure GxS can also be detected by the approaches [Diff_5e-8_], $\left[{\mathrm{Strat}}_{1\mathrm{e-}5}]\to [{\mathrm{Diff}}_{\alpha_{\mathrm{Diff}}}\right]$ or $\left[{\mathrm{Joint}}_{1\mathrm{e-}5}]\to [{\mathrm{Diff}}_{\alpha_{\mathrm{Diff}}}\right]$, but with less power compared to $\left[{\mathrm{Overall}}_{1\mathrm{e-}5}]\to [{\mathrm{Diff}}_{\alpha_{\mathrm{Diff}}}\right]$ (18.9%, 56.2%, and 51.% compared to 85.3% for a quantitative GxS with $R_{1}^{2}$ = 0.167%, $R_{2}^{2}$ = 0.042%). In all scenarios, $\left[{\mathrm{Overall}}_{1\mathrm{e-}5}\to {\mathrm{Diff}}_{\alpha_{\mathrm{Diff}}}\right]$ is the best approach to detect pure GxS (with 0.7%, 80.8%, and >99.9% power for the three effect sizes of R_1_ depicted in **Fig 2A–2C**, respectively) and [Diff_5e-8_] is the best approach to detect a qualitative GxS with equal effects pointing into opposite directions (43.7%, >99%, and >99% power for the three effect sizes of R_1_ depicted in **Fig 2A–2C**, respectively).

Among the two-stage approaches, $\left[{\mathrm{Joint}}_{1\mathrm{e-}5}]\to {[\mathrm{Diff}}_{\alpha_{\mathrm{Diff}}}\right]$ has more power compared to $\left[{\mathrm{Strat}}_{1\mathrm{e-}5}]\to [{\mathrm{Diff}}_{\alpha_{\mathrm{Diff}}}\right]$ for qualitative GxS and similar power for pure and quantitative GxS. However, both two-stage approaches are outperformed in all scenarios by one of the one-stage approaches [Diff_5e-8_] and $\left[{\mathrm{Overall}}_{1\mathrm{e-}5}\to {\mathrm{Diff}}_{\alpha_{\mathrm{Diff}}}\right]$ (**Fig 2A–2C**).

In summary, the difference test without any filtering, [Diff_5e-8_], and the approach filtering for overall association followed by the difference test, $\left[{\mathrm{Overall}}_{1\mathrm{e-}5}\to {\mathrm{Diff}}_{\alpha_{\mathrm{Diff}}}\right]$, are the two best approaches to detect qualitative or pure/quantitative GxS, respectively.

### Influence of varying filtering thresholds

We next investigate how the filtering threshold impacts the power of the approaches to identify GxS. We thus compute the power analytically for the various approaches under the same scenarios as before (balanced strata design, *n*_*1*_ = 100,000, *n*_*2*_ = 100,000), but now vary the filtering threshold, *α*_*Filter*_, from 0.05 to 5 x 10^−8^, again with a Bonferroni-corrected α-level for the consecutive difference test, 0.05/*M*, with *M* being the number of filtered variants.

**Fig 3:**
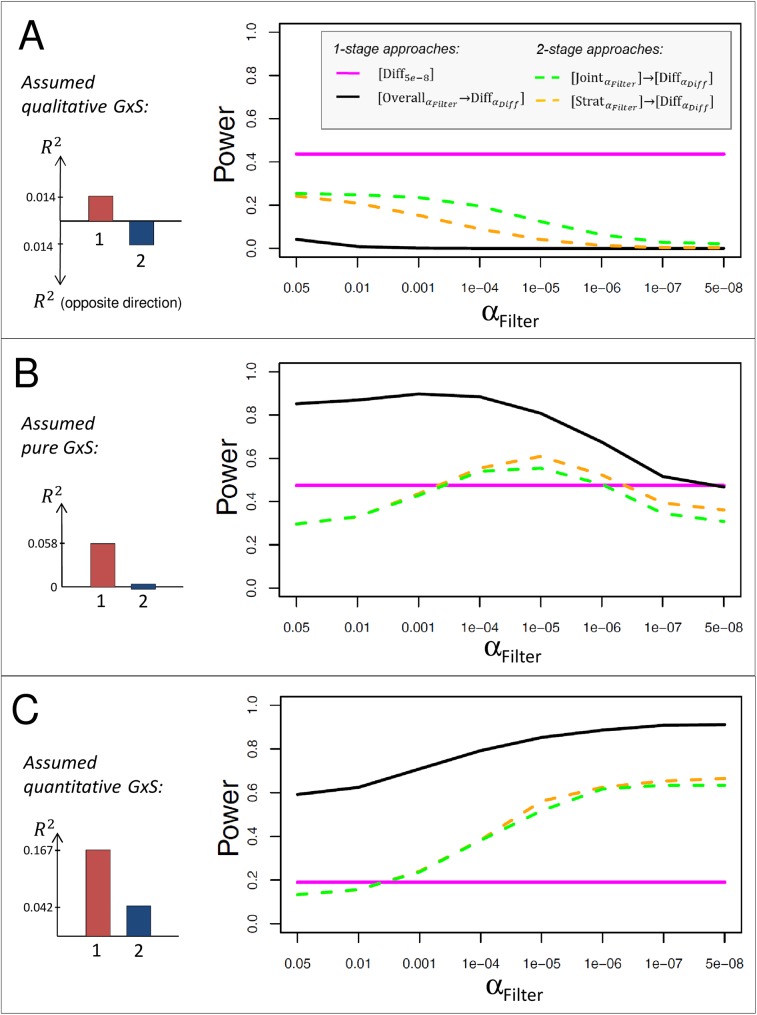
Influence of filtering threshold on the power to detect GxS. Shown is the power to detect GxS for the same approaches as in **Fig 2** (*n*_*1*_ = 100,000, *n*_*2*_ = 100,000), but here with varying filtering thresholds and fixed $R_{2}^{2}$ relative to $R_{1}^{2}$ for different types of GxS: A. qualitative GxS with small stratum-specific effects ($R_{1}^{2}=0.014\%,\;R_{2}^{2}=0.014\%$ into opposite direction), B. pure GxS with medium sized stratum 1 effect ($R_{1}^{2}=0.058\%,\;R_{2}^{2}=0\%$), and C. quantitative GxS with large stratum 1 and smaller stratum 2 effect ($R_{1}^{2}=0.167\%,\;R_{2}^{2}=0.042\%$ into the same direction). The effect sizes for stratum 1 are chosen as those observed for the WHRadjBMI loci around *STAB1*, *PPARG*, *or LYPLAL1*. The power of [Diff_5e-8_] is constant due to the lack of any filtering.

We observe the following: (1) For qualitative GxS (**Fig 3A**), the difference test without filtering, [Diff_5e-8_], shows better power than any filtering approach, irrespective of α_Filter_. (2) For pure GxS (**Fig 3B**), the overall association filtering, $\left[{\mathrm{Overall}}_{\alpha_{\mathrm{Filter}}}\to {\mathrm{Diff}}_{\alpha_{\mathrm{Diff}}}\right]$, has the best power, irrespective of α_Filter_; the power of this approach is the highest for α_Filter_ of 0.05 to 1x10^-4^, but then decreases with decreasing α_Filter_ down to a level that coincides with the power of the difference test without filtering (power = 46.7% and 47.4%, for approaches $\left[{\mathrm{Overall}}_{5\mathrm{e-}8}\to {\mathrm{Diff}}_{\alpha_{\mathrm{Diff}}}\right]$ and [Diff_5e-8_], respectively). The two-stage approaches $\left[{\mathrm{Joint}}_{\alpha_{\mathrm{Filter}}}]\to {[\mathrm{Diff}}_{\alpha_{\mathrm{Diff}}}\right]$ and $\left[{\mathrm{Strat}}_{\alpha_{\mathrm{Filter}}}]\to {[\mathrm{Diff}}_{\alpha_{\mathrm{Diff}}}\right]$ show a maximum power at *α*_*Filter*_ = 1x10^-5^. (3) For quantitative GxS (**Fig 3C**), the overall association filtering, $\left[{\mathrm{Overall}}_{\alpha_{\mathrm{Filter}}}\to {\mathrm{Diff}}_{\alpha_{\mathrm{Diff}}}\right]$, again, has the highest power of all approaches, irrespective of α_Filter_. The power of all three filtering approaches increases with decreasing filtering threshold.

Altogether, while [Diff_5e-8_] outperforms all filtering approaches for qualitative GxS, $\left[{\mathrm{Overall}}_{\alpha_{\mathrm{Filter}}}\to {\mathrm{Diff}}_{\alpha_{\mathrm{Diff}}}\right]$ is most powerful for pure/quantitative GxS. This approach can benefit from less stringent filtering (i.e., larger *α*_*Filter*_, larger M) to detect pure GxS, but from more stringent filtering (i.e., smaller *α*_*Filter*_, smaller M) to detect quantitative GxS, requiring a compromise to serve both.

### Influence of unbalanced strata designs

We next investigate how an unbalanced strata design impacts the power of the approaches to identify GxS. We compute the power analytically for the same scenarios and approaches as previously, but now we model unbalanced strata designs by varying the proportion of the stratum sample sizes (with a total *n* = 200,000, as before). Denoting *f* = *n*_*2*_*/n*_*1*_, with stratum 1 defining the stratum with the larger effect, *f* = 0.05 indicates that stratum 1 (with the larger effect) is 20 times larger than stratum 2, whereas *f* = 20 indicates that stratum (with the larger effect) is in very small with only a 20^th^ of stratum 2 sample size.

**Fig 4:**
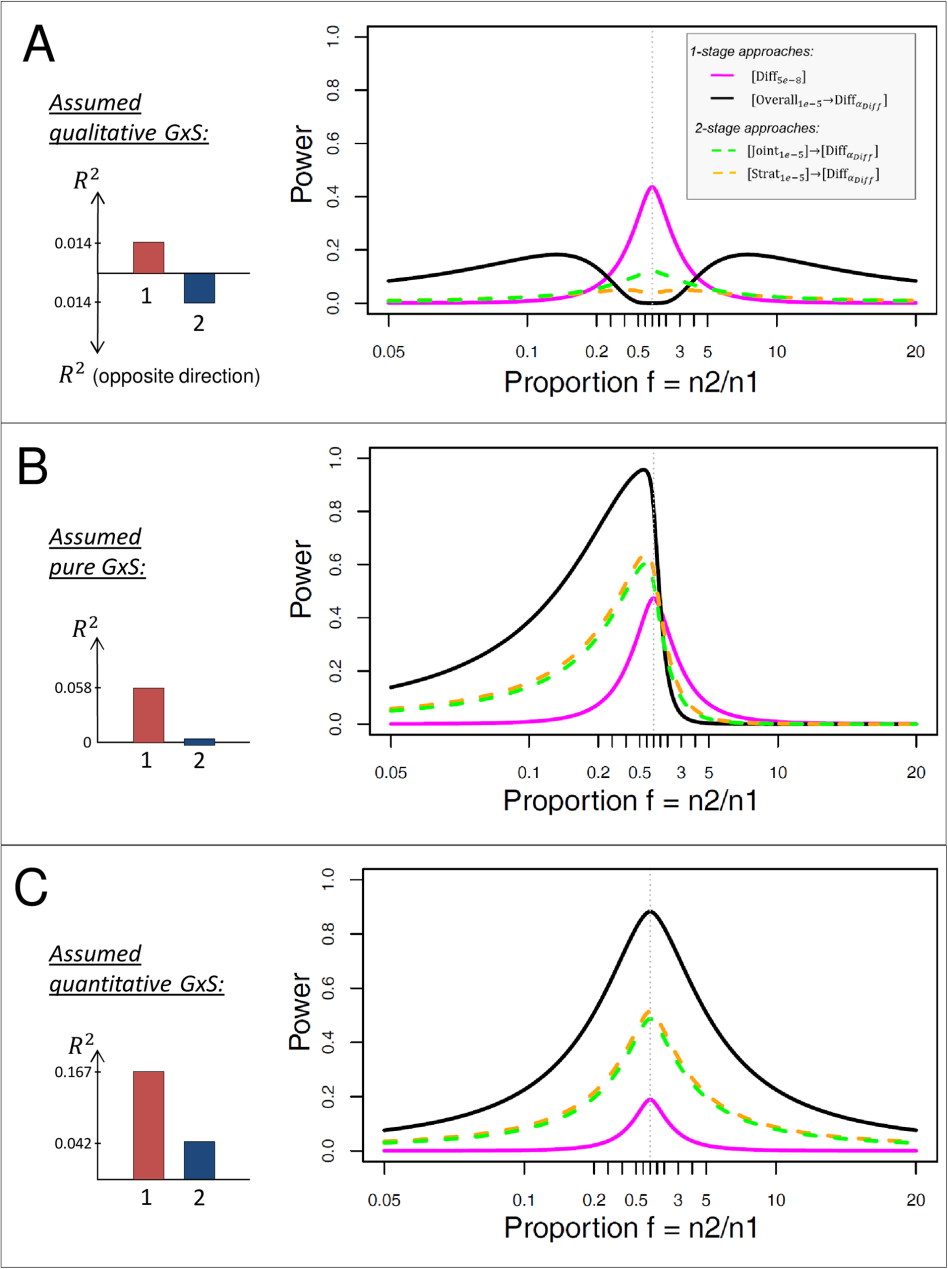
Power of stratified GWAMA approaches to identify GxS for unbalanced strata design. Shown is the power to detect GxS for the same approaches as in **Fig 2**, but here for unbalanced strata designs with varying proportion of stratum sample sizes, *f = n*_*2*_*/n*_*1*_, with stratum 1 being the one with the larger effect. Effect sizes are chosen as **in Fig 3** (fixed $R_{2}^{2}$ relative to $R_{1}^{2},\;R_{1}^{2}$ as observed for WHRadjBMI loci around *STAB1*, *PPARG*, *or LYPLAL1*): A. qualitative GxS with small $R_{1}^{2}$ ($R_{1}^{2}=0.014\%,\;R_{2}^{2}=0.014\%$, into opposite direction), B. pure GxS with medium $R_{1}^{2}$ ($R_{1}^{2}=0.058\%,\;R_{2}^{2}=0\%$), and C. quantitative GxS with large $R_{1}^{2}$ ($R_{1}^{2}=0.167\%,\;R_{2}^{2}=0.042\%$).

As expected from theory, we find that, for all three types of GxS (**Fig 4A–4C**), the power of [Diff_5e-8_] is symmetric to and at a maximum at *f* = 1. This indicates that the difference test without filtering is most efficient, if the two strata are balanced in size, and that its power does not depend on whether the larger effect is in the larger or in the smaller stratum.

For a qualitative GxS (**Fig 4A**), [Diff_5e-8_] shows the best power for moderately unbalanced strata designs (0.2 < *f* < 5), whereas $\left[{\mathrm{Overall}}_{1\mathrm{e-}5}\to {\mathrm{Diff}}_{\alpha_{\mathrm{Diff}}}\right]$ shows best power for more extremely unbalanced strata designs (*f* < 0.2 or *f* > 5). Here, power curves for all approaches are symmetric to *f* = 1, because absolute genetic effects are the same across strata. However, the symmetry of the filtered approaches disappears when varying *R*_*2*_ (**S6–S8 Figs**).

For a pure GxS (**Fig 4B**) with the effect in the larger stratum (*f* < 1), the filtering approaches $\left[{\mathrm{Overall}}_{1\mathrm{e-}5}\to {\mathrm{Diff}}_{\alpha_{\mathrm{Diff}}}\right]$, $\left[{\mathrm{Strat}}_{1\mathrm{e-}5}]\to {[\mathrm{Diff}}_{\alpha_{\mathrm{Diff}}}\right]$, and $\left[{\mathrm{Joint}}_{1\mathrm{e-}5}]\to {[\mathrm{Diff}}_{\alpha_{\mathrm{Diff}}}\right]$ have larger power than the difference test alone with a maximum power at *f* ~ 0.66 (i.e. ‘effect’ stratum 1 is 1.5-times larger than the ‘no effect’ stratum 2). The best approach here is the overall filtering approach, $\left[{\mathrm{Overall}}_{1\mathrm{e-}5}\to {\mathrm{Diff}}_{\alpha_{\mathrm{Diff}}}\right]$. When the effect is in the smaller stratum (*f* > 1), the difference test without filtering, [Diff_5e-8_], can provide a power gain over the filtering approaches: For the presented scenario, the power of [Diff_5e-8_] surpasses the power of the filtering approaches at *f* ~ 1.5 (‘no effect’ stratum 2 is 1.5 times larger than the ‘effect’ stratum 1). Generally, when using the filtering approaches, it is easier to identify pure GxS with the effect in the larger stratum (*f* < 1) than with the effect in the smaller stratum (*f* > 1), while the difference test alone does not depend on whether the effect is in the smaller or the larger stratum.

For quantitative GxS (**Fig 4C**) and for the presented scenario, the power of all approaches is symmetric to and at maximum at *f* = 1. Irrespective of *f*, $\left[{\mathrm{Overall}}_{1\mathrm{e-}5}\to {\mathrm{Diff}}_{\alpha_{\mathrm{Diff}}}\right]$ displays the best power to identify quantitative GxS compared to all other considered approaches (**Fig 4C, S6–S8 Figs**).

Altogether, $\left[{\mathrm{Overall}}_{1\mathrm{e-}5}\to {\mathrm{Diff}}_{\alpha_{\mathrm{Diff}}}\right]$ is the most powerful approach to detect pure/quantitative GxS, for all stratum designs. It has also the best power to detect effects pointing into opposite directions (qualitative difference) when the strata are extremely unbalanced. [Diff_5e-8_] is the most powerful approach to detect qualitative GxS, when the sample sizes of the strata do not differ too extremely.

### Application to real sex-stratified GWAMA results for waist-hip ratio

We exemplify the impact of our approaches on the number of identifiable sexually dimorphic loci for WHRadjBMI, based on our sex-stratified GWAMA results from the GIANT consortium (up to 77,000 men and 98,000 women) [12].

First, in order to derive empirical type I error based on the real GIANT data, we apply all seven approaches to the real sex-stratified meta-analyzed GWAMA results and evaluate lambda factors and QQ plots. For [Diff_5e-8_] and $\left[{\mathrm{Overall}}_{\alpha_{\mathrm{Filter}}}\to {\mathrm{Diff}}_{\alpha_{\mathrm{Diff}}}\right]$, we observe no inflation of difference P-Values (*λ*_*GC*_ = 1.02 and *λ*_*GC*_ = 1.06, respectively, **S9 Fig**). However, we observe inflated difference P-values for the one-stage approaches $\left[{\mathrm{Strat}}_{\alpha_{\mathrm{Filter}}}\to {\mathrm{Diff}}_{\alpha_{\mathrm{Diff}}}\right]$ and $\left[{\mathrm{Joint}}_{\alpha_{\mathrm{Filter}}}\to {\mathrm{Diff}}_{\alpha_{\mathrm{Diff}}}\right]$, (*λ*_*GC*_ = 6.67 and *λ*_*GC*_ = 6.39, respectively, **S9 Fig**). This is in-line with the statistical theory and our results from simulated data, which support the notion that the stratified and the alternative joint tests depend on the difference test, while the overall test appears to be independent.

Next, in order to evaluate the detectability of sexually dimorphic loci for WHRadjBMI, we apply the four approaches [Diff_5e-8_], $\left[{\mathrm{Overall}}_{1\mathrm{e-}5}\to {\mathrm{Diff}}_{\alpha_{\mathrm{Diff}}}\right]$, $\left[{\mathrm{Strat}}_{1\mathrm{e-}5}]\to [{\mathrm{Diff}}_{\alpha_{\mathrm{Diff}}}\right]$, and $\left[{\mathrm{Joint}}_{1\mathrm{e-}5}]\to [{\mathrm{Diff}}_{\alpha_{\mathrm{Diff}}}\right]$ to the real sex-stratified meta-analyzed GWAMA results. When accounting for the subtle relatedness (r = 0.05 for one-stage, r = 0.03 for stage 2 of the two-stage approaches), we identify a total of 10 independent loci with significant sex-difference across all considered approaches (**Table 2, S2 Table and S3 Table**). The 10 loci include one qualitative, six pure and three quantitative sex-differences. Consistent with our power computations, the one-stage approaches identify more loci than the two-stage approaches, the qualitative difference is only detected by [Diff_5e-8_] and $\left[{\mathrm{Overall}}_{1\mathrm{e-}5}\to {\mathrm{Diff}}_{\alpha_{\mathrm{Diff}}}\right]$ identifies all pure and quantitative differences (nine loci). The overall association test filtering at an *α*_*Filter*_ of 5 x 10^−8^, $\left[{\mathrm{Overall}}_{5\mathrm{e-}8}\to {\mathrm{Diff}}_{\alpha_{\mathrm{Diff}}}\right]$, identifies 8 of these and one additional; this approach is, in fact, the approach used by Locke and colleagues for BMI [25] and by Shungin and colleagues for WHRadjBMI [5], when only the genome-wide significant loci with main genetic effect are tested for interaction. When applying the two approaches [Diff_5e-8_] and $\left[{\mathrm{Overall}}_{1\mathrm{e-}5}\to {\mathrm{Diff}}_{\alpha_{\mathrm{Diff}}}\right]$ jointly, 10 sexually dimorphic loci for WHRadjBMI are detected in the GIANT data. When ignoring the subtle relatedness, we detect only 9 loci with significant sex-difference across all considered approaches (the one missed with a sex-difference P-value of 9.1 x 10^−8^ instead of 4.1 x 10^−8^ when accounting for the relatedness).

**Table 2:** Application to real sex-stratified GWAMA data for WHRadjBMI from the GIANT GENDER project. Shown are the 10 identified loci with GxSex by each approach (‘x’ indicating that the locus was identified by the respective approach) at a Bonferroni-corrected significance level, based on the GIANT data for WHRadjBMI (up to 77,000 men and 98,000 women) [12]. Detailed association results are provided in **S2 Table** for the one-stage approaches and in **S3 Table** for the two-stage approaches.

		One-stage approaches		Two-stage approaches	
Locus	Type	[Diff_5e-8_]	$\left[{\mathrm{Overall}}_{1\mathrm{e-}5}\to {\mathrm{Diff}}_{\alpha_{\mathrm{Diff}}}\right]$	$\left[{\mathrm{Strat}}_{1\mathrm{e-}5}]\to [{\mathrm{Diff}}_{\alpha_{\mathrm{Diff}}}\right]$	$\left[{\mathrm{Joint}}_{1\mathrm{e-}5}]\to [{\mathrm{Diff}}_{\alpha_{\mathrm{Diff}}}\right]$
*LRRC69*	Qualitative	x	-	-	-
*SLC30A10*	Pure	x	x	x	x
*COBLL1*	Pure	x	x	x	x
*NKX3-1*	Pure	-	x	-	-
*PLXND1*	Pure	x	x	-	-
*PPARG*	Pure	-	x	-	-
*TNFAIP8*	Pure	-	x	-	-
*ADAMTS9*	Quantitative	-	x	x	x
*ITPR2*	Quantitative	-	x	-	-
*VEGFA*	Quantitative	x	x	x	x

In summary, our application of the approaches to real sex-stratified GWAMA data for WHRadjBMI corroborates our simulation-based and analytical evaluations of type I error rates and power.

## Discussion

### Recommendations

**Fig 5:**
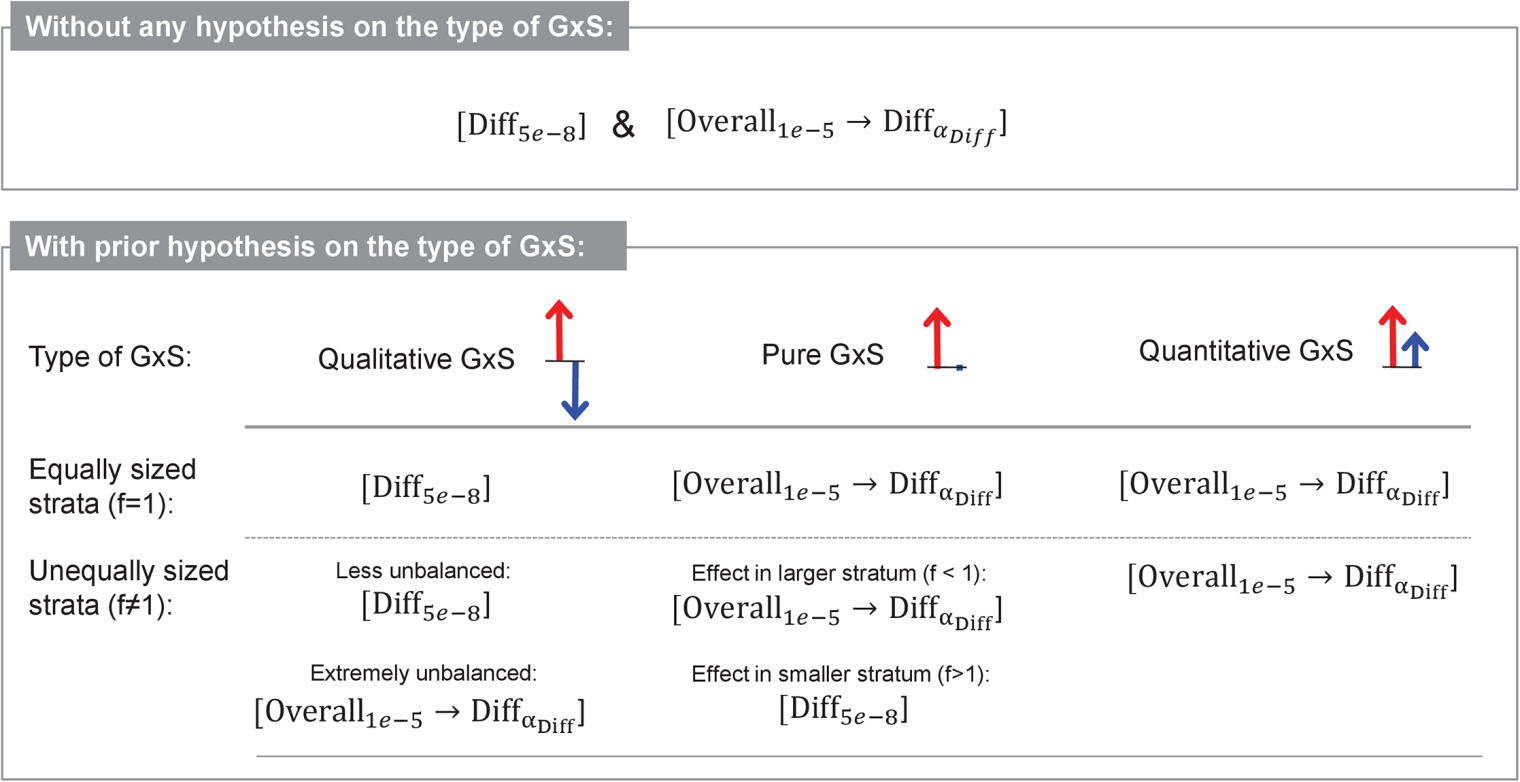
Recommended stratified GWAMA approaches to detect GxS. Shown are the recommended approaches to detect GxS when there is no prior hypothesis on the type of GxS (*H*_*0*_: *No GxS*) and when there is a prior hypothesis on the type of GxS (*H*_*0*_: *No qualitative GxS*; or *H*_*0*_: *No pure GxS*; or *H*_*0*_: *No quantitative GxS*). The recommendations vary on the degree to which the strata sample sizes differ (*f* being the proportion of stratum 2 sample size over stratum 1 sample size, *f* = *n*_*2*_*/n*_*1*_ with stratum 1 being the stratum with the larger absolute value of the genetic effect).

Based on our evaluations of type I error and power, we found that two of the approaches to search for genetic effects with GxS based on stratified GWAMA data keep type I error control and are the most powerful: the genome-wide difference test and the overall association filtering prior to the difference test. Which of these two performed better than the other, depended on the type of GxS (qualitative, pure or quantitative) and on the strata design (balanced, unbalanced). We thus provide a recommendation of the best approach depending on the type of strata-difference and strata design (**Fig 5**). Generally, for any stratified GWAMA project that aims at detecting genetic variants with GxS without any hypothesis on the specific type of GxS and irrespective of the strata design, we recommend to perform two approaches in parallel: (i) a genome-wide screen for difference testing at an α-level of 5 x 10^-8^, and (ii) an approach that filters for overall association at *P*_*Overall*_ < 10^−5^ and then tests this subset of genetic variants for difference at a Bonferroni-corrected α-level.

This recommendation is based on several findings of our comparisons: (1) The difference test without filtering, [Diff_5e-8_], has the best power to detect qualitative GxS in most scenarios and the overall association test filtering prior to the difference test, $\left[{\mathrm{Overall}}_{\alpha_{\mathrm{Filter}}}\to {\mathrm{Diff}}_{\alpha_{\mathrm{Diff}}}\right]$, has the best power for pure/quantitative GxS with few exceptions. (2) The approaches filtering for stratified or alternative joint association prior to difference testing in the same set of GWAMA results (one-stage approaches $\left[{\mathrm{Strat}}_{\alpha_{\mathrm{Filter}}}\to {\mathrm{Diff}}_{\alpha_{\mathrm{Diff}}}\right]$ and $\left[{\mathrm{Joint}}_{\alpha_{\mathrm{Filter}}}\to {\mathrm{Diff}}_{\alpha_{\mathrm{Diff}}}\right]$) violate type I error. Since stratified or (alternative) joint association tests are commonly applied for filtering in GWAMA literature [8,12,26,27], it is important to note that testing the selected variants for GxS necessitates an independent set of GWAMA results (two stage approaches $\left[{\mathrm{Strat}}_{\alpha_{\mathrm{Filter}}}]\to {[\mathrm{Diff}}_{\alpha_{\mathrm{Diff}}}\right]$ and $\left[{\mathrm{Joint}}_{\alpha_{\mathrm{Filter}}}]\to {[\mathrm{Diff}}_{\alpha_{\mathrm{Diff}}}\right]$). (3) The two-stage approaches were outperformed by at least one one-stage approach. The reason for this is that splitting the data into two artificial stages does not make use of the total sample size neither for the filtering nor for the difference test, which is in-line with previous work[28]. (4) We found that the filtering threshold of the overall association test had a substantial impact on power. Since a less stringent filtering (larger *α*_*Filter*_, more variants selected) yielded larger power to detect pure GxS, while more stringent filtering (smaller alpha, fewer variants selected) yielded larger power for quantitative GxS, a compromise is required. We found a filtering threshold of *P*_*Overall*_ < 10^−5^ to work well in most scenarios.

Several aspects are interesting to note: No matter what filtering approach is used, it is easier to identify GxS with the larger effect in the larger stratum as compared to a GxS with the larger effect is in the smaller stratum. For example, a filtering approach can be expected to detect more variants with the predominant effect in non-smokers rather than in smokers (assuming more non-smokers in the data and same number of genetic effects specific to non-smokers compared to smokers). The genome-wide difference screen has no preference towards an effect in the larger or in the smaller stratum, but it loses power with increasing imbalance of strata.

It should also be noted that the commonly used GWAMA approach to screen for genome-wide significant overall association (*P*_*Overall*_ < 5 x 10^-8^) and to subsequently test identified lead variants for difference between strata performs well to find pure and quantitative difference. The question here remains, whether many genetic effects with opposite effect direction in the two strata exist (qualitative GxS) that are missed by such an approach or whether such effects are biologically plausible. Without the utilization of approaches with sufficient power to detect qualitative GxS, this question will be left unanswered.

### Strengths and limitations

We focused here on approaches to identify GxS that are directly applicable to stratified GWAMAs results. We assumed a dichotomous stratification variable and continuous outcome that follow identical normal distributions. To our knowledge, this is the first study to evaluate such approaches systematically and to provide recommendations on how to design a GWAMA that aims to identify genetic variants with between-strata differences.

Despite the fact that our work is comprehensive and covers numerous approaches and scenarios, there are still scenarios that we have not considered in order to stay focused. This includes binary outcomes, different phenotypic variances between strata, between-study differences of the genetic effect, or a measurement error in the phenotype that differs between studies and strata. Still, our results can readily be translated into stratified GWAMAs of binary outcomes using logistic regression and different phenotypic variances can be implemented by extending the power formulae. Between-study heterogeneity of genetic effects and measurement error issues will be important extensions, but are widely ignored in the current GWAMA approaches to locus identification.

The stratified GWAMA approaches can be translated into interaction GWAMA approaches where the interaction term is fitted per study and meta-analyzed (**S4 Table**). Particularly the difference test from the stratified approach is equivalent to a test of the interaction term when there is no interaction between covariates and the strata S and that the trait variance is the same in the two strata. Our results and recommendations based on stratified GWAMAS can be transferred to interaction modeling and suggest a parallel approach for testing interaction genome-wide and a filtering for overall association (main effect) prior to testing the interaction effect. The analogy suggests that a joint test filtering (testing jointly the main and the interaction effect) with subsequently testing selected variants for interaction in the same set of GWAMA results violates type I error. The stratified GWAMA framework has some important advantages and disadvantages compared to an interaction GWAMA framework (see **S2 Note** for a detailed discussion of pros and cons of the stratified and the interaction GWAMA frameworks). The focus on stratified GWAMA results here was motivated by the much easier logistics of computing stratum-specific GWAS and the meta-analysis of stratum-specific genetic effects compared to interaction modeling in GWAS and the respective meta-analysis.

We did not consider GxE interaction methods that are based on linearly increasing phenotypic variance [29], meta-regression of summary statistics [30], or on case-only [31], data adaptive [32,33], empirical Bayes [34] or other 2-step methods that involve filtering on gene-exposure or gene-strata association [35–38]. The latter rely on the assumption of independence of implemented steps [39] and any methods that involve case-only designs or empirical Bayes methods are limited to binary disease outcome. The reason for not extending our considerations to the noted methods was that they either involve data sets that are not directly available from the stratified GWAMA or that they were only described for single genome-wide interaction studies rather than meta-analysis settings or for binary outcomes. Their implementation and transferability into a stratified GWAMA setting with continuous outcome may be limited and are as yet unclear.

Finally, by reducing the filtered variants to the independent lead variants (e.g. variant with smallest filtering test P-value within +/- 500kB), we might miss the correlated variant close-by that is the truly interesting variant with between-stratum difference (i.e. the variant with the smallest difference test P-value). We could extend to approaches that select all variants meeting the filtering threshold without restricting to lead variants in this step, subsequently testing all selected variants for difference and alternative approaches for multiple testing that can handle correlated variants, such as employing a Bonferroni-correction based on the effective number of independent variants [23,40] or a false-discovery rate approach [41].

### Summary and conclusion

In summary, we recommend the genome-wide difference test without filtering, [Diff_5e-8_], to search for genetic effects that point into opposite direction in two strata and the overall association test filtering prior to a difference test, $\left[{\mathrm{Overall}}_{\alpha_{\mathrm{Filter}}}\to {\mathrm{Diff}}_{\alpha_{\mathrm{Diff}}}\right]$, to detect genetic effects that are only or more pronounced in one stratum. When there is no hypothesis on the type of GxS that is aimed to identify, we recommend applying these two approaches in parallel. For the overall association test filtering, a filtering threshold of 1 x 10^−5^ appears to be reasonable, while a filtering threshold of 5 x 10^-8^ is equivalent to the common GWAMA approach to identify variants with genome-wide significance and test only these variants for GxS.

Our results provide guidelines for current and future GWAMAs that aim at the identification of genetic effects with GxS. By these clear recommendations, researchers will be more motivated to search for GxS and by enhancing our searches with the most powerful approaches we will be able to unravel GxS for complex disease. Ultimately, our knowledge of genetic effects that show differential effects between strata will help our understanding of how a variant exerts its effect on the disease outcome under study.

## Supporting information

S1 Methods Defining lead variants and the number of independent loci filtered. (DOCX)

S2 Methods Details on the simulation-based evaluation of type I error. (DOCX)

S3 Methods Derivation of analytical power formulae for the considered statistical tests and approaches. (DOCX)

S4 Methods Details on the considered scenarios of analytical power computations. (DOCX)

S1 Note Effect of differences in allele frequency between strata. (DOCX)

S2 Note Comparison of stratified and interaction GWAMA frameworks. (DOCX)

S3 Note GIANT consortium authors. (DOCX)

S1 TableStatistical tests for stratified GWAMA approaches to identify GxS.Stated are the tests that can be applied based on the meta-analyzed stratum-specific genetic effect estimates, ${\hat{\beta }}_{i}$, and standard errors, se_i_ (i = 1,2), the respective null hypotheses, test statistics, nomenclature for P-values and the usage.(DOCX)

S2 TableTen loci with significant sex-difference for WHRadjBMI identified by the one-stage approaches.The table shows the lead variants identified by the two one-stage approaches [Diff_*5e-8*_] and $\left[{\mathrm{Overall}}_{1\mathrm{e-}5}\to {\mathrm{Diff}}_{\alpha_{\mathrm{Diff}}}\right]$ that were applied to the sex-stratified GWAMA results for WHRadjBMI (up to 77,000 men and 98,000 women) from the GIANT consortium [12]. Significant sex-difference P-Values (corrected for correlation between strata using r = 0.05 as estimated from the GIANT data on 77,000 men and 98,000 women) are marked in bold. Sex-difference P-Values (uncorrected for correlation) were added to the table for comparison.(DOCX)

S3 TableFour loci with significant sex-difference for WHRadjBMI identified by the two-stage approaches.The table shows the lead variants identified by the two-stage approaches $\left[{\mathrm{Strat}}_{1\mathrm{e-}5}]\to [{\mathrm{Diff}}_{\alpha_{\mathrm{Diff}}}\right]$ and $\left[{\mathrm{Joint}}_{1\mathrm{e-}5}]\to [{\mathrm{Diff}}_{\alpha_{\mathrm{Diff}}}\right]$ that were applied to the sex-stratified GWAMA results for WHRadjBMI (up to 35,000 and 42,000 men; and up to 43,000 and 55,000 women in the two stages respectively; from the GIANT consortium [12]. Significant stage 2 sex-difference P-Values (corrected for correlation between strata using r = 0.03 as estimated from the Stage 2 GIANT data on 42,000 men and 55,000 women) are marked in bold. Stage 2 sex-difference P-Values (uncorrected for correlation) were added to the table for comparison.(DOCX)

S4 TableStatistical tests for interaction GWAMA approaches to identify GxS.Instead of applying two stratified linear regression models per study and meta-analyzing stratified genetic estimates, an interaction GWAMA framework involves one interaction model per study, $Y=\propto_{ik}+\beta_{G_{ik}}G_{ik}+\beta_{S_{ik}}S_{ik}+\beta_{{GxS}_{ik}}G_{ik}xS_{ik}+E_{ik}^{\left(j\right)}$, where S codes strata membership (i.e. S = 0 for stratum 1, S = 1 for stratum 2). Meta-analyzed genetic main effects (${\hat{\beta }}_{G}$) and gene-strata interaction effects (${\hat{\beta }}_{GxS}$) with corresponding standard errors (*se*_*G*_ and *se*_*GxS*_) are obtained from study-specific genetic main effects (${\hat{\beta }}_{G_{ik}}$) or gene-strata interaction (${\hat{\beta }}_{{GxS}_{ik}}$) effects, respectively. Stated are the tests that can be applied based on the interaction GWAMA framework, the respective null hypotheses, test statistics, nomenclature for P-values and the usage.(DOCX)

S1 Fig Seven stratified GWAMA approaches to identify GxS. The figure visualizes the approach without filtering as well as the approaches with filtering. The filtering approaches can either be conducted as one-stage or as two-stage approaches. For the one-stage approaches, the filtering and the difference test are applied to one large stratified GWAMA result of total sample size *N* (blue). For the two-stage approaches, the filtering and the difference test are applied consecutively to two independent stratified GWAMA results of size *N/2* (purple and orange).
(TIF)

S2 FigSimulation-based evaluation of type 1 error for one-stage approaches assuming balanced strata.Shown are the difference P-values simulated under the null hypotheses of no GxS given no stratum-specific effects ($H_{0}^{\beta =0}$) or of no GxS given identical stratum-specific effects ($H_{0}^{\beta \neq 0}$) for the one-stage approaches $\left[{\mathrm{Diff}}_{\alpha_{\mathrm{Diff}}}\right]$, $\left[{\mathrm{Overall}}_{\alpha_{\mathrm{Filter}}}\to {\mathrm{Diff}}_{\alpha_{\mathrm{Diff}}}\right]$, $\left[{\mathrm{Strat}}_{\alpha_{\mathrm{Filter}}}\to {\mathrm{Diff}}_{\alpha_{\mathrm{Diff}}}\right]$ and $\left[{\mathrm{Joint}}_{\alpha_{\mathrm{Filter}}}\to {\mathrm{Diff}}_{\alpha_{\mathrm{Diff}}}\right]$. The QQ plots are based on simulated phenotypes and simulated genotypes to reflect A) $H_{0}^{\beta =0}$ with *MAF* = 0.05, B) $H_{0}^{\beta =0}$ with *MAF* = 0.30, C) $H_{0}^{\beta \neq 0}$ with *MAF* = 0.05, and D) $H_{0}^{\beta \neq 0}$ with *MAF* = 0.30. We here assume α_Filter_ = 0.05 for $H_{0}^{\beta =0}$ and *α*_*Filter*_ = 10^−5^ for $H_{0}^{\beta \neq 0}$ and two equally sized (balanced) strata (100,000 individuals in each stratum, *f* = 1).(TIF)

S3 FigSimulation-based evaluation of type 1 error for two-stage approaches assuming balanced strata.Shown are the simulated difference P-values for the two-stage approaches $\left[{\mathrm{Overall}}_{\alpha_{\mathrm{Filter}}}]\to {[\mathrm{Diff}}_{\alpha_{\mathrm{Diff}}}\right]$, $\left[{\mathrm{Strat}}_{\alpha_{\mathrm{Filter}}}]\to {[\mathrm{Diff}}_{\alpha_{\mathrm{Diff}}}\right]$ and $\left[{\mathrm{Joint}}_{\alpha_{\mathrm{Filter}}}]\to [{\mathrm{Diff}}_{\alpha_{\mathrm{Diff}}}\right]$. We here assume two equally sized (balanced) strata (50,000 individuals in each stratum and stage, *f* = 1). Results are presented for varying MAF and null hypotheses as in **S2 Fig**.(TIF)

S4 FigSimulation-based evaluation of type 1 error for one-stage approaches assuming unbalanced strata.Shown are simulated difference P-values for the one-stage approaches $\left[{\mathrm{Diff}}_{\alpha_{\mathrm{Diff}}}\right]$, $\left[{\mathrm{Overall}}_{\alpha_{\mathrm{Filter}}}\to {\mathrm{Diff}}_{\alpha_{\mathrm{Diff}}}\right]$, $\left[{\mathrm{Strat}}_{\alpha_{\mathrm{Filter}}}\to {\mathrm{Diff}}_{\alpha_{\mathrm{Diff}}}\right]$ and $\left[{\mathrm{Joint}}_{\alpha_{\mathrm{Filter}}}\to {\mathrm{Diff}}_{\alpha_{\mathrm{Diff}}}\right]$. We here assume two unbalanced strata (66,000 and 134,000 individuals in the two strata, respectively, *f* = 0.33 and *f* = 3). Results are presented for varying MAF and null hypotheses as in **S2 Fig**.(TIF)

S5 FigSimulation-based QQ-plots for two-stage approaches and unbalanced strata designs.Shown are simulated difference P-values for the two-stage approaches $\left[{\mathrm{Overall}}_{\alpha_{\mathrm{Filter}}}]\to {[\mathrm{Diff}}_{\alpha_{\mathrm{Diff}}}\right]$, $\left[{\mathrm{Strat}}_{\alpha_{\mathrm{Filter}}}]\to {[\mathrm{Diff}}_{\alpha_{\mathrm{Diff}}}\right]$ and $\left[{\mathrm{Joint}}_{\alpha_{\mathrm{Filter}}}]\to [{\mathrm{Diff}}_{\alpha_{\mathrm{Diff}}}\right]$. We here assume two unbalanced sized strata (33,000 and 67,000 individuals in the two strata and in each stage, *f* = 0.33 and *f* = 3). Results are presented for varying MAF and null hypotheses as in **S2 Fig**.(TIF)

S6 FigPower of stratified GWAMA approaches to identify GxS assuming unbalanced strata and a small effect in stratum 1.Shown is the power to detect GxS for the same approaches and designs as in **Fig 3** (unbalanced strata designs with varying proportion of stratum sample sizes, f = n_2_/n_1_, with stratum 1 being the one with the larger effect). Effect size in stratum 1 is fixed to $R_{1}^{2}=0.014\%$, as observed for the small WHRadjBMI effect at *STAB1*. The effect in stratum 2 is fixed to A. 0.014%, into opposite direction (qualitative GxS; same as main **Fig 3A**), B. $R_{2}^{2}=0.007\%$, into opposite direction (qualitative GxS), C. $R_{2}^{2}=0.003\%$, into opposite direction (qualitative GxS). D. $R_{2}^{2}=0\%$ (pure GxS), E. $R_{2}^{2}=0.003\%$, into consistent direction (quantitative GxS), and F. $R_{2}^{2}=0.007\%$, into consistent direction (quantitative GxS).(TIF)

S7 FigPower of the stratified GWAMA approaches to identify GxS assuming unbalanced strata design and a medium effect in stratum 1.Shown is the power to detect GxS for the same approaches and designs as in **Fig 3** (unbalanced strata designs with varying proportion of stratum sample sizes, f = n_2_/n_1_, with stratum 1 being the one with the larger effect). Effect size in stratum 1 is fixed to $R_{1}^{2}=0.058\%$, as observed for the medium WHRadjBMI effect at *PPARG*. The effect in stratum 2 is fixed to A. 0.058%, into opposite direction (qualitative GxS), B. $R_{2}^{2}=0.029\%$, into opposite direction (qualitative GxS), C. $R_{2}^{2}=0.014\%$, into opposite direction (qualitative GxS). D. $R_{2}^{2}=0\%$ (pure GxS; same as main **Fig 3B**), E. $R_{2}^{2}=0.014\%$, into consistent direction (quantitative GxS), and F. $R_{2}^{2}=0.029\%$, into consistent direction (quantitative GxS).(TIF)

S8 FigPower of the stratified GWAMA approaches to identify GxS assuming unbalanced strata design and a large effect in stratum 1.Shown is the power to detect GxS for the same approaches and designs as in **Fig 3** (unbalanced strata designs with varying proportion of stratum sample sizes, f = n_2_/n_1_, with stratum 1 being the one with the larger effect). Effect size in stratum 1 is fixed to $R_{1}^{2}=0.0.167\%$, as observed for the large WHRadjBMI effect at *LYPLAL1*. The effect in stratum 2 is fixed to A. 0.167%, into opposite direction (qualitative GxS), B. $R_{2}^{2}=0.084\%$, into opposite direction (qualitative GxS), C. $R_{2}^{2}=0.042\%$, into opposite direction (qualitative GxS). D. $R_{2}^{2}=0\%$ (pure GxS), E. $R_{2}^{2}=0.042\%$, into consistent direction (quantitative GxS, same as main **Fig 3C**), and F. $R_{2}^{2}=0.084\%$, into consistent direction (quantitative GxS).(TIF)

S9 FigQQ plots showing the results of the application of one-stage approaches to sex-stratified GWAMA from the GIANT consortium.The QQ plot contrasts observed and expected difference P-Values for the considered 1-stage approaches $\left[{\mathrm{Diff}}_{\alpha_{\mathrm{Diff}}}\right]$, $\left[{\mathrm{Overall}}_{0.05}\to {\mathrm{Diff}}_{\alpha_{\mathrm{Diff}}}\right]$, $\left[{\mathrm{Strat}}_{0.05}\to {\mathrm{Diff}}_{\alpha_{\mathrm{Diff}}}\right]$ and $\left[{\mathrm{Joint}}_{0.05}\to {\mathrm{Diff}}_{\alpha_{\mathrm{Diff}}}\right]$ obtained from an application of approaches to real sex-stratified GWAMA data for WHRadjBMI from the GIANT consortium.(TIF)
