# Supplementary material for: Approaches to detect genetic effects that differ between two strata in genome-wide meta-analyses: Recommendations based on a systematic evaluation
Source: PLoS One. 2017 Jul 27;12(7):e0181038. doi: 10.1371/journal.pone.0181038 (PMC5531538; doi:10.1371/journal.pone.0181038)
Supplement: S1 Methods — (DOCX) [file pone.0181038.s001.docx]

# S1 Methods. Defining lead variants and the number of independent loci filtered

When we select the variants using any of the three tests for filtering at a given filtering threshold *α_Filter_*, the selected variants usually contain multiple variants that are correlated. As we need to control for multiple testing in the next step, when we apply the difference test to the selected variants, we limit the difference testing to one lead variant per locus and apply a Bonferroni-corrected α-level on the number of independent lead. Commonly used criteria to define a locus are LD-based (e.g., including all variants with pairwise *r^2^* > 0.2 to the variant with the smallest filtering test P-value) or distance-based (e.g., including all variants *±*500Kb from the variant with the smallest filtering test P-value). We here apply a distance-based criterion, *±* 500Kb and define the variant with the lowest filtering-test P-Value within one locus as the lead-variant. Thus, the independent lead-variants are those that are put forward and tested for difference. The number of selected lead variants thus defines the number of independent difference tests performed: this number is denoted as *M*. To correct for the multiple testing of *M* independent lead variants, we utilize a Bonferroni-corrected α-level, *α_Diff_ = 0.05/M.*

To derive a realistic *M* for the power computations, instead of making assumptions on the distribution of real effect sizes genome-wide, we obtain the number of variants filtered by each of the approaches using the GIANT GENDER data [[1](#_ENREF_1)] for balanced strata designs (f = 1) and the GIANT SMOKING data [[2](#_ENREF_2)] for unbalanced strata design with (reflecting f~3.0 or 0.33).

# References

1. Randall JC, Winkler TW, Kutalik Z, Berndt SI, Jackson AU, et al. (2013) Sex-stratified genome-wide association studies including 270,000 individuals show sexual dimorphism in genetic loci for anthropometric traits. PLoS Genet 9: e1003500.

2. Justice AE, Winkler TW, Feitosa MF, Graff M, Fisher VA, et al. (2017) Genome-wide meta-analysis of 241,258 adults accounting for smoking behaviour identifies novel loci for obesity traits. Nat Commun 8: 14977.
