# Supplementary material for: Approaches to detect genetic effects that differ between two strata in genome-wide meta-analyses: Recommendations based on a systematic evaluation
Source: PLoS One. 2017 Jul 27;12(7):e0181038. doi: 10.1371/journal.pone.0181038 (PMC5531538; doi:10.1371/journal.pone.0181038)
Supplement: S2 Methods — (DOCX) [file pone.0181038.s002.docx]

# S2 Methods. Details on the simulation-based evaluation of Type I Error

For each considered simulation scenario, we sample genotypes from *G ~ B(2, MAF)* and assign phenotypes based on *Y ~ N(0,1)* for $H_{0}^{\beta=0}$, or based on *Y|G=0 ~ N(0,1)*, *Y|G=1 ~ N(b,1)* and *Y|G=2 ~ N(2b,1)* for $H_{0}^{\beta\neq0}$, modeling an additive genetic effect. We then split the 200,000 individuals into two strata of 100,000 individuals each (for balanced design) or 66,000 and 134,000 (for unbalanced designs, *f* = 0.33 and *f* = 3); we split these further into two equally sized stages for the two-stage approaches. We repeat the procedure 1,000,000 times, thus generating 1,000,000 independent genetic variants and compute stratum-specific genetic effect estimates $\hat{\beta}_{i,l}$ with standard errors ${se}_{i,l}$ (*l* = 1…1,000,000; *i* = 1, 2). We then apply the seven stratified GWAMA approaches to identify GxS: We test $\hat{\beta}_{1,l}$ and $\hat{\beta}_{2,l}$ for between-strata difference (no filtering) or apply one of the three filtering tests in the full (one-stage) or the first stage data (two-stages). For each of the six filtering approaches, we select *M* variants with varying filtering thresholds and test these for difference in the same (one-stage) or the second stage data (two stages).
