# Supplementary material for: Approaches to detect genetic effects that differ between two strata in genome-wide meta-analyses: Recommendations based on a systematic evaluation
Source: PLoS One. 2017 Jul 27;12(7):e0181038. doi: 10.1371/journal.pone.0181038 (PMC5531538; doi:10.1371/journal.pone.0181038)
Supplement: S3 Methods — (DOCX) [file pone.0181038.s003.docx]

# S3 Methods. Derivation of analytical power formulae for the considered statistical tests and approaches

In the following, we provide the analytical power formulae for the four considered statistical tests (the difference test, overall association test, test for stratified association, and the alternative joint test). The formulae are written in dependence of the α-level of the test, sample size in stratum 1, *n_1,_* the ratio of stratum 2 sample size to stratum 1 sample size, *f* = *n_2_/n_1_*, and the stratum-specific genetic effect measures, $R_{1}^{2}$ and $R_{2}^{2}$, as introduced in the methods of the main text in formula (2). Furthermore, the standard error of the stratum-specific genetic effect *β_i_* can be written in dependency of $R_{i}^{2}$ and *n_i_* as

| ${{se}_{i}}^{2}=\left[ \left( 1-R_{i}^{2} \right)\sigma_{Y}^{2} \right]/\left( n_{i}\sigma_{G}^{2} \right)$ | (3), |
| --- | --- |

where, $\sigma_{Y}^{2}$ is the stratum-specific phenotypic variance (assumed to be identical across strata) and $\sigma_{G}^{2}$ is the genotypic variance (assumed to be identical across strata) [[1](#_ENREF_1)].

*Power for the difference test*: The power of the *difference test* (**Supplementary Table 1**) to detect a difference between the stratum-specific beta-estimates, $\hat{\beta}_{1}$ and $\hat{\beta}_{2}$, is obtained as the power of a Z test making use of the equations (2) and (3):

| ${Power}_{Diff}\left( \alpha,R_{1}^{2},R_{2}^{2},n_{1},f \right)==\Phi\left( -z_{1-\frac{\alpha}{2}}-\sqrt{n_{1}}\frac{R_{1}-R_{2}}{\sqrt{1-R_{1}^{2}+\frac{1}{f}(1-R_{2}^{2})}} \right)+\Phi\left( -z_{1-\frac{\alpha}{2}}+\sqrt{n_{1}}\frac{R_{1}-R_{2}}{\sqrt{1-R_{1}^{2}+\frac{1}{f}(1-R_{2}^{2})}} \right)$ | (4). |
| --- | --- |

Here, $\Phi$ denotes the cumulative standard normal distribution and $z_{q}$ the q-th quantile of $\Phi$.

*Power for the stratified test*: Under the assumption of independent subjects not only within strata, but also across strata, the power of the *stratified test* to detect any non-null stratum-specific effect, $\beta_{1}\neq0$ or $\beta_{2}\neq0$, is given by

| ${Power}_{Strat}\left( \alpha,R_{1}^{2},R_{2}^{2},n_{1},f \right)=$  $={Power}_{z}\left( \frac{\alpha}{2},R_{1}^{2},n_{1} \right)+{Power}_{z}\left( \frac{\alpha}{2},R_{2}^{2},fn_{1} \right)-{Power}_{z}\left( \frac{\alpha}{2},R_{1}^{2},n_{1} \right){Power}_{z}\left( \frac{\alpha}{2},R_{2}^{2},fn_{1} \right)$ | (5), |
| --- | --- |

where ${Power}_{z}(\alpha,R_{i}^{2},n_{i})$ denotes the power of a stratum-specific z test depending on alpha, the stratum-specific effect measure $R_{i}^{2}$ and the stratum-specific sample size *n_i_*, here as the power of a z-test in stratum 1 (*R_1_, n_1_*) and in stratum 2 (*R_2_, n_2_*). ${Power}_{z}(\alpha,R_{i}^{2},n_{i})$ is obtained by making use of the equations (2) and (3):

| ${Power}_{z}\left( \alpha,R_{i}^{2},n_{i} \right)=\Phi\left( -z_{1-\frac{\alpha}{2}}-\sqrt{\frac{n_{i}R_{i}^{2}}{1-R_{i}^{2}}} \right)+\Phi\left( -z_{1-\frac{\alpha}{2}}+\sqrt{\frac{n_{i}R_{i}^{2}}{1-R_{i}^{2}}} \right)$ | (6). |
| --- | --- |

*Power for the overall test*: The power of the *overall test* (**Supplementary Table 1**) to detect stratum-combined effects is obtained by utilizing equations (2) and (3) with the power of a regular z test:

| ${Power}_{Overall}\left( \alpha,R_{1}^{2},R_{2}^{2},n_{1},f \right)==\Phi\left( -z_{1-\frac{\alpha}{2}}-\sqrt{n_{1}} \frac{\frac{R_{1}}{(1-R_{1}^{2})}+\frac{fR_{2}}{(1-R_{2}^{2})}}{\sqrt{\frac{1}{1-R_{1}^{2}}+\frac{f}{(1-R_{2}^{2})}}} \right)+\Phi\left( -z_{1-\frac{\alpha}{2}}+\sqrt{n_{1}} \frac{\frac{R_{1}}{(1-R_{1}^{2})}+\frac{fR_{2}}{(1-R_{2}^{2})}}{\sqrt{\frac{1}{1-R_{1}^{2}}+\frac{f}{(1-R_{2}^{2})}}} \right)$ | (1). |
| --- | --- |

*Power for the alternative joint test:* The power of the alternative *joint test* is given by

| ${Power}_{Joint}\left( \alpha,R_{1}^{2},R_{2}^{2},n_{1},f \right)=1-X_{2,\lambda\left( R_{1}^{2},R_{2}^{2},n_{1},f \right)}^{2}\left( \chi_{2,1-\alpha} \right)$ | (2), |
| --- | --- |

Here, $\chi_{2,q}$ is the q-th quantile of a chi-square distribution with 2df, $X_{2,\lambda}^{2}$ the cumulative distribution function of a non-central chi-square distribution with 2df and non-centrality parameter$\lambda$ , which can be calculated as

| $\lambda\left( R_{1}^{2},R_{2}^{2},n_{1},f \right)=\frac{n_{1}R_{1}^{2}}{1-R_{1}^{2}}+\frac{fn_{1}R_{2}^{2}}{1-R_{2}^{2}}$ | (3). |
| --- | --- |

*Power for the considered approaches to identify GxS*

There is only one approach without filtering, which is the difference test, which is applied in a one-stage design, $\text{[}\text{Diff}_{\alpha_{Diff}}\text{]}$. Its power to detect a true GxS is given by ${Power}_{Diff}$ (see (4)).

For approaches that involve any filtering, the Bayes formula can be applied and the power to detect a true GxS (i.e., true H_A_: β_1_ ≠ β_2_) can be computed as

$${Power}_{Approach}=Prob\left( P_{Filter}<\alpha_{Filter}, P_{Diff}<\alpha_{Diff}|H_{A} \right).$$

If the two tests are independent or applied to two different data sets, this simplifies to

$${Power}_{Approach}=Prob\left( P_{Filter}<\alpha_{Filter}|H_{A} \right)\cdot Prob\left( P_{Diff}<\alpha_{Diff}|H_{A} \right)$$

$= {Power}_{Filter}\cdot{Power}_{Diff}$.

Here, *α_Filter_* _,_ *P_Filter_* and *Power_Filter_* denote the filtering threshold, P-value and power of the filtering test (overall, joint, or stratified test), and *α_Diff_* _,_ *P_Diff_* and *Power_Diff_* denote the α-level, P-Value and power of the difference test.

# References

1. Rosner B (2006) Fundamentals of biostatistics. Belmont, CA: Thomson-Brooks/Cole. xix, 868 p. p.
