# Supplementary material for: Approaches to detect genetic effects that differ between two strata in genome-wide meta-analyses: Recommendations based on a systematic evaluation
Source: PLoS One. 2017 Jul 27;12(7):e0181038. doi: 10.1371/journal.pone.0181038 (PMC5531538; doi:10.1371/journal.pone.0181038)
Supplement: S4 Methods — (DOCX) [file pone.0181038.s004.docx]

# S4 Methods. Details on the considered scenarios of analytical power computations

We vary strata designs (f = 0.5 to 20) and split the 200,000 accordingly. We further split stratum-specific sample sizes equally for two-stage approaches. We assume realistic effect sizes in stratum 1: a small, medium or large effect as observed for rs6784615, rs4684854, or rs2820443, respectively (near *STAB1, PPARG*, or *LYPLAL1,* variance explained=0.014%, 0.058% or 0.167%), for waist-hip-ratio adjusted for BMI (WHRadjBMI) in the GIANT data([1](#_ENREF_1)). We model varying types of GxS (qualitative, pure, quantitative) and vary the effect size in the second stratum, R_2_, relative to R_1_ (|R_2_|<=|R_1_|). For the difference test without filtering, we apply a genome-wide significance level, *α_Diff_* = 5 x 10^-8^. For approaches with filtering, we vary the filtering threshold (*α_Filter_* =0.05 to 5 x 10^-8^), on which the number of variants with a subsequent difference test, *M*, and thus the significance level, α_Diff_ = 0.05/M, depends. To derive realistic M, instead of making assumptions on the distribution of real effect sizes genome-wide, we obtain the number of variants filtered by each of the approaches using publically available GIANT data ([1](#_ENREF_1), [2](#_ENREF_2)).

# References

1. Randall JC, Winkler TW, Kutalik Z, Berndt SI, Jackson AU, Monda KL, et al. Sex-stratified genome-wide association studies including 270,000 individuals show sexual dimorphism in genetic loci for anthropometric traits. PLoS genetics. 2013 Jun;9(6):e1003500. PubMed PMID: 23754948. Pubmed Central PMCID: 3674993.

2. Justice AE, Winkler TW, Feitosa MF, Graff M, Fisher VA, Young K, et al. Genome-wide meta-analysis of 241,258 adults accounting for smoking behaviour identifies novel loci for obesity traits. Nature communications. 2017 Apr 26;8:14977. PubMed PMID: 28443625. Pubmed Central PMCID: 5414044.
