# Supplementary material for: Approaches to detect genetic effects that differ between two strata in genome-wide meta-analyses: Recommendations based on a systematic evaluation
Source: PLoS One. 2017 Jul 27;12(7):e0181038. doi: 10.1371/journal.pone.0181038 (PMC5531538; doi:10.1371/journal.pone.0181038)
Supplement: S1 Note — (DOCX) [file pone.0181038.s005.docx]

# S1 Note. Assumption of similar allele frequencies across strata

Our evaluation is based on the assumption of similar allele frequencies without restriction of generality due to the following reasons.

First, non-significant differences in allele frequencies (e.g., to a genome-wide significance level) reflect random differences. Consequently, allele frequencies in the population can be considered as equal (our assumption).

Second, significant differences in allele frequencies would imply that the genetic variant G and the exposure variable E are correlated, which is very rare as environmental factors have low heritability (unless they are ancestry related). Along with an effect of G on the outcome Y, E either acts as confounder, as mediator or as collider.

A confounding effect of E in genetics would derive from population stratification, which is accounted for by each study. We would assume there is no other confounding effect for genetics.

A mediator E disappears in the regression model by adjusting for E (which we do through the stratification by E). If E is a perfect mediator, there would be no effect in either stratum and hence no GxE interaction.

All other cases, e.g. partial mediation or collider, can give rise to artefactual associations (due to collider bias, heteroscedasticity, etc.), which need further scrutiny. For these reasons we suggest that in case when G and E are significantly associated (i.e. the allele frequency is significantly different across strata) and a significant interaction result is obtained, these artefacts are closely examined.

In summary, different allele frequencies across strata are rare and when present they can give rise to spurious and hence problematic GxE interactions. For these reasons we can assume "without restriction of generality" that the allele frequencies are equal and variants violating this assumption are safer to be excluded from further analysis.
