# Supplementary material for: Approaches to detect genetic effects that differ between two strata in genome-wide meta-analyses: Recommendations based on a systematic evaluation
Source: PLoS One. 2017 Jul 27;12(7):e0181038. doi: 10.1371/journal.pone.0181038 (PMC5531538; doi:10.1371/journal.pone.0181038)
Supplement: S2 Note — (DOCX) [file pone.0181038.s006.docx]

# S2 Note. Comparison of stratified and interaction GWAMA frameworks

Our considered stratified GWAMA framework has some advantages and disadvantages compared to an interaction GWAMA framework where the interaction term is fitted directly per study and meta-analyzed across studies.

One disadvantage of the stratified GWAMA framework is that it is principally limited to dichotomous environmental factors S. However, extension to ordinal exposure is straight forward: one can run meta-analyses separately for each category and use trend tests to compare genetic effects across categories. Still, such methods are not yet available for genome-wide category-specific summary statistics. In order to make it applicable to continuous exposure, the exposure variable can be dichotomized into two groups by applying a specific threshold. Although this dichotomization loses information, there are also scenarios where utilizing the dichotomized exposure is inevitable or preferable. These include scenarios where modelling interaction with the continuous variable implies unclear application or scenarios where the dichotomized exposure fits the reality better than its continuous form. For example, due to a decline in hormonal levels, women change body shape specifically after menopause and fitting an interaction model with dichotomized age (e.g. comparing the younger vs those older than 50 years of age) might be beneficial over using continuous age.

Another disadvantage of the stratified GWAMA framework is that it cannot properly model relatedness across strata. For example, brothers and sisters would be analyzed separately in a sex-stratified GWAMA, a circumstance that may inflate overall or alternative joint test associations and deflate the sex-difference statistics (i.e., sex-specific genetic effect estimates would be less different under the null due to relatedness across sexes). Although some correction for this issue has been described for the difference test ([1](#_ENREF_1)) and for the overall association test ([2](#_ENREF_2)), a systematic methodological evaluation is lacking and a similar correction for the alternative joint test is not yet available.

One advantage of the stratified GWAMA framework is that the identified GxS are not confounded by covariate-strata interactions (CxS): In the stratified GWAMA framework, if a covariate is added to the model and the model is applied separately by stratum, any CxS is accounted for. In contrast, in the interaction GWAMA framework, a covariate is often included into the models without a CxS term, which does not always effectively remove the effect of the confounder C ([3](#_ENREF_3)). However, if the stratification variable is correlated with the covariate, the identified GxS may be confounded by gene-covariate interactions (GxC). Admittedly both, the stratified and the interaction GWAMA framework can be affected by the latter if GxC terms are left out of the respective regression models.

Finally, a strong advantage of the stratified GWAMA framework is the potentially larger total sample size and thus larger power compared to the interaction GWAMA framework, especially in the context of large-scale GWAMA consortia that combine data from hundreds of GWAS. The main reason for this are studies that lack individuals for a specific stratum. While such studies can generally contribute to the stratified GWAMA, they have to be excluded from the interaction GWAMA, because the gene-strata interaction cannot be modelled on the study level. For example, the sex-stratified GWAMA in the GIANT consortium from 2013 involved >7,000 men from five men-only studies and >30,000 women from six women-only studies that would have been missed by an interaction GWAMA framework ([1](#_ENREF_1)). On the other hand, stratified GWAMA analysis requires sensitivity analysis to exclude cohort-specific effects. A further reason for the larger sample size of the stratified GWAMA is that it does not require specialized GWAS tools as compared to fitting an interaction term genome-wide, which makes it more easily applicable for study analysts.

# References

1. Randall JC, Winkler TW, Kutalik Z, Berndt SI, Jackson AU, Monda KL, et al. Sex-stratified genome-wide association studies including 270,000 individuals show sexual dimorphism in genetic loci for anthropometric traits. PLoS genetics. 2013 Jun;9(6):e1003500. PubMed PMID: 23754948. Pubmed Central PMCID: 3674993.

2. Zhu X, Feng T, Tayo BO, Liang J, Young JH, Franceschini N, et al. Meta-analysis of correlated traits via summary statistics from GWASs with an application in hypertension. American journal of human genetics. 2015 Jan 8;96(1):21-36. PubMed PMID: 25500260. Pubmed Central PMCID: 4289691.

3. Keller MC. Gene x environment interaction studies have not properly controlled for potential confounders: the problem and the (simple) solution. Biological psychiatry. 2014 Jan 01;75(1):18-24. PubMed PMID: 24135711. Pubmed Central PMCID: 3859520.
