# Supplementary material for: Approaches to detect genetic effects that differ between two strata in genome-wide meta-analyses: Recommendations based on a systematic evaluation
Source: PLoS One. 2017 Jul 27;12(7):e0181038. doi: 10.1371/journal.pone.0181038 (PMC5531538; doi:10.1371/journal.pone.0181038)
Supplement: S3 Note — (DOCX) [file pone.0181038.s007.docx]

**S3 Note. GIANT Consortium authors.**

The following list of GIANT consortium members is based on the author list from Randall et al. 2013 (PMID 23754948):

Joshua C Randall*^1,2^, Thomas W Winkler*^3^, Zoltán Kutalik*^4,5^, Sonja I Berndt*^6^, Anne U Jackson^7^, Keri L Monda^8^, Tuomas O Kilpeläinen^9^, Tõnu Esko^10,11^, Reedik Mägi^2,10^, Shengxu Li^9,12^, Tsegaselassie Workalemahu^13^, Mary F Feitosa^14^, Damien C Croteau-Chonka^15^, Felix R Day^9^, Tove Fall^16^, Teresa Ferreira^2^, Stefan Gustafsson^16^, Adam E Locke^7^, Iain Mathieson^2^,Andre Scherag^17^, Sailaja Vedantam^18,19,20^, Andrew R Wood^21^, Liming Liang^22,23^, Valgerdur Steinthorsdottir^24^, Gudmar Thorleifsson^24^, Emmanouil T Dermitzakis^25^, Antigone S Dimas^2,25,26^, Fredrik Karpe^27^, Josine L Min^2^, George Nicholson^28,29^, Deborah J Clegg^30^, Thomas Person^30^, Jon P Krohn^2^,Sabrina Bauer^31^, Christa Buechler^31^, Kristina Eisinger^31^, DIAGRAM Consortium, Amélie Bonnefond^32^, Philippe Froguel^33,32^, MAGIC Investigators,Jouke-Jan Hottenga^34^, Inga Prokopenko^2,27^, Lindsay L Waite^35^, Tamara B Harris^36^, Albert Vernon Smith^37,38^, Alan R Shuldiner^39,40^, Wendy L McArdle^41^, Mark J Caulfield^42^, Patricia B Munroe^42^, Henrik Grönberg^16^, Yii-Der Ida Chen^43,44^, Guo Li^45^, Jacques S Beckmann^46,4^, Toby Johnson^4,5,42^, Unnur Thorsteinsdottir^24,47^, Maris Teder-Laving^10^, Kay-Tee Khaw^48^, Nicholas J Wareham^9^, Jing Hua Zhao^9^, Najaf Amin^49^, Ben A Oostra^50,51,52^, Aldi T Kraja^14^, Michael A Province^14^, L Adrienne Cupples^53^, Nancy L Heard-Costa^54^, Jaakko Kaprio^55,56,57^, Samuli Ripatti^57,1,58^, Ida Surakka^57,58^, Francis S Collins^59^, Jouko Saramies^60^, Jaakko Tuomilehto^61,62,63,64^, Antti Jula^65^, Veikko Salomaa^66^, Jeanette Erdmann^67,68^, Christian Hengstenberg^69^, Christina Loley^68,70^, Heribert Schunkert^67,68^, Claudia Lamina^71^, H.- Erich Wichmann^72,73^, Eva Albrecht^74^, Christian Gieger^74^, Andrew A Hicks^75^, Åsa Johansson^76,77^, Peter P Pramstaller^75,78,79^, Sekar Kathiresan^80,81,82^, Elizabeth K Speliotes^83,84^, Brenda Penninx^85^, Anna-Liisa Hartikainen^86^, Marjo-Riitta Jarvelin^87,88,89,90^, Ulf Gyllensten^76^, Dorret I Boomsma^34^, Harry Campbell^91^, James F Wilson^91^, Stephen J Chanock^6^, Martin Farrall^92^, Anuj Goel^92^, Carolina Medina-Gomez^49,93,52^, Fernando Rivadeneira^49,93,52^, Karol Estrada^49,93,52^, André G Uitterlinden^49,93,52^, Albert Hofman^49,52^, M Carola Zillikens^93,52^, Martin den Heijer^94^, Lambertus A Kiemeney^95,96,97^, Andrea Maschio^98^, Per Hall^16^, Jonathan Tyrer^99^, Alexander Teumer^100^, Henry Völzke^101^, Peter Kovacs^102^, Anke Tönjes^103,104^, Massimo Mangino^105^, Tim D Spector^105^, Caroline Hayward^106^, Igor Rudan^91^, Alistair S Hall^107^, Nilesh J Samani^108,109^, Antony Paul Attwood^1,110^, Jennifer G Sambrook^110,111^, Joseph Hung^112,113^, Lyle J Palmer^114,115^, Marja-Liisa Lokki^116^, Juha Sinisalo^117^, Gabrielle Boucher^118^, Heikki Huikuri^119^, Mattias Lorentzon^120^, Claes Ohlsson^120^, Niina Eklund^58,11^, Johan G Eriksson^121,122,123^, Cristina Barlassina^124^, Carlo Rivolta^4^, Ilja M Nolte^125^, Harold Snieder^125,126^, Melanie M Van der Klauw^127,126^, Jana V Van Vliet-Ostaptchouk^127,126^, Pablo V Gejman^128,129^, Jianxin Shi^6^, Kevin B Jacobs^6,130^, Zhaoming Wang^6,130^, Stephan JL Bakker^131^, Irene Mateo Leach^132^, Gerjan Navis^131^, Pim van der Harst^132,133^, Nicholas G Martin^134^, Sarah E Medland^134^, Grant W Montgomery^135^, Jian Yang^136^, Daniel I Chasman^137,138^, Paul M Ridker^137,138^, Lynda M Rose^137^, Terho Lehtimäki^139^, Olli Raitakari^140,141^, Devin Absher^35^, Carlos Iribarren^142^, Hanneke Basart^143^, Kees G Hovingh^143^, Elina Hyppönen^144^, Chris Power^144^, Denise Anderson^145,146^, John P Beilby^113,147,148^, Jennie Hui^113,149,148,147^, Jennifer Jolley^110^, Hendrik Sager^150^, Stefan R Bornstein^151^, Peter EH Schwarz^151^, Kati Kristiansson^57,58^, Markus Perola^57,58,10^, Jaana Lindström^63^, Amy J Swift^59^, Matti Uusitupa^152,153^, Mustafa Atalay^154^, Timo A Lakka^155,154^, Rainer Rauramaa^155,156^, Jennifer L Bolton^91^, Gerry Fowkes^91^, Ross M Fraser^91^, Jackie F Price^91^, Krista Fischer^10^, Kaarel KrjutÅ¡kov^10^, Andres Metspalu^10^, Evelin Mihailov^10,11^, Claudia Langenberg^9,157^, Jian'an Luan^9^, Ken K Ong^9,158^, Peter S Chines^59^, Sirkka M Keinanen-Kiukaanniemi^159,160^, Timo E Saaristo^161,162^, Sarah Edkins^1^, Paul W Franks^163,164,165^, Göran Hallmans^165^, Dmitry Shungin^163,165,166^, Andrew David Morris^167^, Colin NA Palmer^167^, Raimund Erbel^168^, Susanne Moebus^17^, Markus M Nöthen^169,170^, Sonali Pechlivanis^17^, Kristian Hveem^171^, Narisu Narisu^59^, Anders Hamsten^172^, Steve E Humphries^173^, Rona J Strawbridge^172^, Elena Tremoli^174^, Harald Grallert^175^, Barbara Thorand^176^, Thomas Illig^175,177^, Wolfgang Koenig^178^, Martina Müller-Nurasyid^179,74,180^, Annette Peters^176^, Bernhard O Boehm^181^, Marcus E Kleber^182,183^, Winfried März^183,184^, Bernhard R Winkelmann^185^, Johanna Kuusisto^186^, Markku Laakso^186^, Dominique Arveiler^187^, Giancarlo Cesana^188^, Kari Kuulasmaa^66^, Jarmo Virtamo^66^, John WG Yarnell^189^, Diana Kuh^158^, Andrew Wong^158^, Lars Lind^190^, Ulf de Faire^191^, Bruna Gigante^191^, Patrik KE Magnusson^16^, Nancy L Pedersen^16^, George Dedoussis^192^, Maria Dimitriou^192^, Genovefa Kolovou^193^, Stavroula Kanoni^1^, Kathleen Stirrups^1^, Lori L Bonnycastle^59^, Inger Njølstad^194^, Tom Wilsgaard^194^, Andrea Ganna^16^, Emil Rehnberg^16^, Aroon Hingorani^157^, Mika Kivimaki^157^, Meena Kumari^157^, Themistocles L Assimes^195^, Inês Barroso^1,196^, Michael Boehnke^7^, Ingrid B Borecki^14^, Panos Deloukas^1^, Caroline S Fox^197^, Timothy Frayling^21^, Leif C Groop^198^, Talin Haritunians^199^, David Hunter^22,13,200^, Erik Ingelsson^16^, Robert Kaplan^201^, Karen L Mohlke^15^, Jeffrey R O'Connell^39^, David Schlessinger^202^, David P Strachan^203^, Kari Stefansson^24,47^, Cornelia M van Duijn^49,52,204^, Goncalo R Abecasis^7^, Mark I McCarthy^2,205,27^, Joel N Hirschhorn^18,19,20^, Lu Qi^13,200^, Ruth JF Loos*^9,206^, Cecilia M Lindgren*^2^, Kari E North*^8^, Iris M Heid*^3,72^

* These authors contributed jointly to this work.

Corresponding for the GIANTconsortium:

Joel N Hirschhorn, **[joelh@broadinstitute.org](mailto:joelh@broadinstitute.org)**

1. Wellcome Trust Sanger Institute, Hinxton, Cambridge, CB10 1SA, UK

2. Wellcome Trust Centre for Human Genetics, University of Oxford, Oxford, OX3 7BN, UK

3. Public Health and Gender Studies, Institute of Epidemiology and Preventive Medicine, Regensburg University Medical Center, Regensburg, Germany

4. Department of Medical Genetics, University of Lausanne, 1005 Lausanne, Switzerland

5. Swiss Institute of Bioinformatics, 1015 Lausanne, Switzerland

6. Division of Cancer Epidemiology and Genetics, National Cancer Institute, National Institutes of Health, Department of Health and Human Services, Bethesda, Maryland 20892, USA

7. Department of Biostatistics, Center for Statistical Genetics, University of Michigan, Ann Arbor, Michigan 48109, USA

8. Department of Epidemiology, School of Public Health, University of North Carolina at Chapel Hill, Chapel Hill, North Carolina 27514, USA

9. MRC Epidemiology Unit, Institute of Metabolic Science, Addenbrooke's Hospital, Cambridge, CB2 0QQ, UK

10. Estonian Genome Center, University of Tartu, Tartu 50410, Estonia

11. Institute of Molecular and Cell Biology, University of Tartu, Tartu 51010, Estonia

12. Department of Epidemiology, Tulane School of Public Health and Tropical Medicine, New Orleans, LA 70112, USA

13. Department of Nutrition, Harvard School of Public Health, Boston, Massachusetts 02115, USA

14. Department of Genetics, Washington University School of Medicine, St Louis, Missouri 63110, USA

15. Department of Genetics, University of North Carolina, Chapel Hill, North Carolina 27599, USA

16. Department of Medical Epidemiology and Biostatistics, Karolinska Institutet, 171 77 Stockholm, Sweden

17. Institute for Medical Informatics, Biometry and Epidemiology (IMIBE), University Hospital of Essen, University of Duisburg-Essen, Essen, Germany

18. Divisions of Genetics and Endocrinology and Program in Genomics, Children's Hospital, Boston, Massachusetts 02115, USA

19. Metabolism Initiative and Program in Medical and Population Genetics, Broad Institute, Cambridge, Massachusetts 02142, USA

20. Department of Genetics, Harvard Medical School, Boston, Massachusetts 02115, USA

21. Genetics of Complex Traits, Peninsula College of Medicine and Dentistry, University of Exeter, Exeter, EX1 2LU, UK

22. Department of Epidemiology, Harvard School of Public Health, Boston, Massachusetts 02115, USA

23. Department of Biostatistics, Harvard School of Public Health, Boston, Massachusetts 02115, USA

24. deCODE Genetics, 101 Reykjavik, Iceland

25. Department of Genetic Medicine and Development, University of Geneva Medical School, Geneva 1211, Switzerland

26. Biomedical Sciences Research Center Al. Fleming, 16672 Vari, Greece

27. Oxford Centre for Diabetes, Endocrinology and Metabolism, University of Oxford, Oxford, OX3 7LJ, UK

28. Department of Statistics, University of Oxford, Oxford OX1 3TG, UK

29. MRC Harwell, Harwell, UK

30. University of Texas Southwestern Medical Center, 5323 Harry Hines Blvd, Dallas Texas 75390-8854

31. Regensburg University Medical Center, Innere Medizin I, 93053 Regensburg, Germany

32. CNRS UMR8199-IBL-Institut Pasteur de Lille, F-59000 Lille, France

33. Department of Genomics of Common Disease, School of Public Health, Imperial College London, W12 0NN, London, UK

34. Department of Biological Psychology, VU University Amsterdam, 1081 BT Amsterdam, The Netherlands

35. Hudson Alpha Institute for Biotechnology, Huntsville, Alabama 35806, USA

36. Laboratory of Epidemiology, Demography, Biometry, National Institute on Aging, National Institutes of Health, Bethesda, Maryland 20892, USA

37. Icelandic Heart Association, Kopavogur, Iceland

38. University of Iceland, Reykjavik, Iceland

39. Department of Medicine, University of Maryland School of Medicine, Baltimore, Maryland 21201, USA

40. Geriatrics Research and Education Clinical Center, Baltimore Veterans Administration Medical Center, Baltimore, Maryland 21201, USA

41. School of Social and Community Medicine, University of Bristol, UK

42. Clinical Pharmacology and Barts and The London Genome Centre, William Harvey Research Institute, Barts and The London School of Medicine and Dentistry, Queen Mary University of London, Charterhouse Square, London EC1M 6BQ, UK

43. Department of OB/GYN and Medical Genetics Institute, Cedars-Sinai Medical Center, Los Angeles, CA

44. Department of Medicine, David Geffen School of Medicine at University of California, Los Angeles, California, USA

45. Cardiovascular Health Research Unit, University of Washington, Seattle, Washington 98101, USA

46. Service of Medical Genetics, Centre Hospitalier Universitaire Vaudois (CHUV) University Hospital, 1011 Lausanne, Switzerland

47. Faculty of Medicine, University of Iceland, 101 Reykjavík, Iceland

48. Department of Public Health and Primary Care, Institute of Public Health, University of Cambridge, Cambridge CB2 2SR, UK

49. Department of Epidemiology, Erasmus MC, Rotterdam, 3015GE, The Netherlands

50. Department of Clinical Genetics, Erasmus MC, Rotterdam, 3015GE, The Netherlands

51. Centre for Medical Systems Biology & Netherlands Consortium on Healthy Aging, Leiden, the Netherlands

52. Netherlands Genomics Initiative (NGI)-sponsored Netherlands Consortium for Healthy Aging (NCHA)

53. Department of Biostatistics, Boston University School of Public Health, Boston, Massachusetts 02118, USA

54. Department of Neurology, Boston University School of Medicine, Boston, Massachusetts 02118, USA

55. National Institute for Health and Welfare, Unit for Child and Adolescent Psychiatry, Helsinki, Finland

56. Finnish Twin Cohort Study, Department of Public Health, University of Helsinki,
00014, Helsinki, Finland

57. Institute for Molecular Medicine Finland (FIMM), University of Helsinki, 00014, Helsinki, Finland

58. National Institute for Health and Welfare, Department of Chronic Disease Prevention, Unit of Public Health Genomics, 00014, Helsinki, Finland

59. Genome Technology Branch, National Human Genome Research Institute, NIH, Bethesda, MD 20892, USA

60. South Karelia Central Hospital, 53130 Lappeenranta, Finland

61. Red RECAVA Grupo RD06/0014/0015, Hospital Universitario La Paz, 28046 Madrid, Spain

62. Centre for Vascular Prevention, Danube-University Krems, 3500 Krems, Austria

63. National Institute for Health and Welfare, Diabetes Prevention Unit, 00271 Helsinki, Finland

64. South Ostrobothnia Central Hospital, 60220 Seinajoki, Finland

65. National Institute for Health and Welfare, Department of Chronic Disease Prevention, Population Studies Unit, 20720 Turku, Finland

66. National Institute for Health and Welfare, Department of Chronic Disease Prevention, Chronic Disease Epidemiology and Prevention Unit, 00271, Helsinki, Finland

67. Nordic Center of Cardiovascular Research (NCCR), 23538 Lübeck, Germany

68. Universität zu Lübeck, Medizinische Klinik II, 23562 Lübeck, Germany

69. Klinik und Poliklinik für Innere Medizin II, Universität Regensburg, 93053 Regensburg, Germany

70. Institut für Medizinische Biometrie und Statistik, Universität zu Lübeck, Universitätsklinikum Schleswig-Holstein, Campus Lübeck, 23562 Lübeck, Germany

71. Division of Genetic Epidemiology, Department of Medical Genetics, Molecular and Clinical Pharmacology, Innsbruck Medical University, 6020 Innsbruck, Austria

72. Institute of Epidemiology I, Helmholtz Zentrum München - German Research Center for Environmental Health, Neuherberg, Germany

73. Institute of Medical Informatics, Biometry and Epidemiology, Chair of Epidemiology, Ludwig-Maximilians-Universität, and Klinikum Grosshadern, Munich, Germany

74. Institute of Genetic Epidemiology, Helmholtz Zentrum München - German Research Center for Environmental Health, 85764 Neuherberg, Germany

75. Center for Biomedicine, European Academy Bozen/Bolzano (EURAC), Bolzano/Bozen, 39100, Italy. Affiliated Institute of the University of Lübeck, Lübeck, Germany.

76. Department of Immunology, Genetics and Pathology, Uppsala University, Sweden

77. Uppsala Clinical Research Center, Uppsala university hospital, Sweden

78. Department of Neurology, General Central Hospital, Bolzano, Italy

79. Department of Neurology, University of Lübeck, Lübeck, Germany.

80. Cardiovascular Research Center and Cardiology Division, Massachusetts General Hospital, Boston, Massachusetts 02114, USA.

81. Center for Human Genetic Research, Massachusetts General Hospital, Boston, Massachusetts 02114, USA.

82. Program in Medical and Population Genetics, Broad Institute of Harvard and Massachusetts Institute of Technology, Cambridge, Massachusetts 02142, USA

83. Center for Computational Medicine and Bioinformatics, University of Michigan, Ann Arbor, Michigan, USA

84. Department of Internal Medicine, Division of Gastroenterology, University of Michigan, Ann Arbor, Michigan, USA

85. Department of Psychiatry, University Medical Centre Groningen, 9713 GZ Groningen, The Netherlands

86. Department of Clinical Sciences/Obstetrics and Gynecology, University of Oulu, 90014 Oulu, Finland

87. Department of Epidemiology and Biostatistics, School of Public Health, Faculty of Medicine, Imperial College London, London, W2 1PG, UK

88. Institute of Health Sciences, University of Oulu, 90014 Oulu, Finland

89. Biocenter Oulu, University of Oulu, 90014 Oulu, Finland

90. National Institute for Health and Welfare, 90101 Oulu, Finland

91. Centre for Population Health Sciences, University of Edinburgh, Teviot Place, Edinburgh, EH8 9AG, Scotland

92. Cardiovascular Medicine, University of Oxford, Wellcome Trust Centre for Human Genetics, Oxford, OX3 7BN, UK

93. Department of Internal Medicine, Erasmus MC, Rotterdam, 3015GE, The Netherlands

94. Department of Internal Medicine, VU University Medical Centre, Amsterdam, The Netherlands

95. Department of Epidemiology, Biostatistics and HTA, Radboud University Nijmegen Medical Centre, 6500 HB Nijmegen, The Netherlands

96. Department of Urology, Radboud University Nijmegen Medical Centre, 6500 HB Nijmegen, The Netherlands

97. Comprehensive Cancer Center East, 6501 BG Nijmegen, The Netherlands

98. Istituto di Neurogenetica e Neurofarmacologia del CNR, Monserrato, 09042, Cagliari, Italy

99. Department of Oncology, University of Cambridge, Cambridge, CB1 8RN, UK

100. Interfaculty Institute for Genetics and Functional Genomics, Ernst-Moritz-Arndt-University Greifswald, 17487 Greifswald, Germany

101. Institute for Community Medicine, Ernst-Moritz-Arndt-University Greifswald, Greifswald, Germany

102. Interdisciplinary Centre for Clinical Research, University of Leipzig, 04103 Leipzig, Germany

103. University of Leipzig, IFB Adiposity Diseases, Leipzig, Germany

104. Department of Medicine, University of Leipzig, 04103 Leipzig, Germany

105. Department of Twin Research and Genetic Epidemiology, King's College London, London, SE1 7EH, UK

106. MRC Human Genetics Unit, Institute for Genetics and Molecular Medicine, Western General Hospital, Edinburgh, EH4 2XU, Scotland, UK

107. Division of Cardiovascular and Neuronal Remodelling, Multidisciplinary Cardiovascular Research Centre, Leeds Institute of Genetics, Health and Therapeutics, University of Leeds, UK

108. Department of Cardiovascular Sciences, University of Leicester, Glenfield Hospital, Leicester, LE3 9QP, UK

109. Leicester NIHR Biomedical Research Unit in Cardiovascular Disease, Glenfield Hospital, Leicester, LE3 9QP, UK

110. Department of Haematology, University of Cambridge, Cambridge CB2 0PT, UK

111. NHS Blood and Transplant, Cambridge Centre, Cambridge, CB2 0PT, UK

112. School of Medicine and Pharmacology, The University of Western Australia, Nedlands WA 6009, Australia

113. Busselton Population Medical Research Foundation Inc., Sir Charles Gairdner Hospital, Nedlands, Western Australia 6009, Australia

114. Genetic Epidemiology and Biostatistics Platform, Ontario Institute for Cancer Research. Toronto, Canada, M5G 1L7

115. Prosserman Centre for Health Research, Samuel Lunenfeld Research Institute, Toronto, Canada, M5G 1X5

116. Transplantation Laboratory, Haartman Institute, University of Helsinki, 00014, Helsinki, Finland

117. Division of Cardiology, Cardiovascular Laboratory, Helsinki University Central Hospital, 00029 Helsinki, Finland

118. Montreal Heart Institute, Montreal, Quebec, H1T 1C8, Canada

119. Institute of Clinical Medicine, Department of Internal Medicine, University of Oulu, 90014 Oulu, Finland

120. Department of Internal Medicine, Institute of Medicine, Sahlgrenska Academy, University of Gothenburg, 413 45 Gothenburg, Sweden

121. Department of General Practice and Primary health Care, University of Helsinki, Helsinki, Finland

122. National Institute for Health and Welfare, 00271 Helsinki, Finland

123. Helsinki University Central Hospital, Unit of General Practice, 00280 Helsinki, Finland

124. University of Milan, Department of Medicine, Surgery and Dentistry, 20139 Milano, Italy

125. Unit of Genetic Epidemiology and Bioinformatics, Dept of Epidemiology, University Medical Center Groningen, University of Groningen, P.O. Box 30001, 9700 RB Groningen, The Netherlands

126. LifeLines Cohort Study, University Medical Center Groningen, University of Groningen, The Netherlands

127. Department of Endocrinology, University Medical Center Groningen, University of Groningen, P.O. Box 30001, 9700 RB Groningen, The Netherlands

128. University of Chicago, Chicago, IL

129. Northshore University Healthsystem, Evanston, Ilinois 60201, USA

130. Core Genotyping Facility, SAIC-Frederick, Inc., NCI-Frederick, Frederick, Maryland 21702, USA

131. Department of Internal Medicine, University Medical Center Groningen, University of Groningen, Groningen

132. Department of Cardiology, University Medical Center Groningen, University of Groningen, The Netherlands

133. Department of Genetics, University Medical Center Groningen, University of Groningen, The Netherlands

134. Genetic Epidemiology Laboratory, Queensland Institute of Medical Research, Queensland 4006, Australia

135. Molecular Epidemiology Laboratory, Queensland Institute of Medical Research, Queensland 4006, Australia

136. Queensland Statistical Genetics Laboratory, Queensland Institute of Medical Research, Queensland 4006, Australia

137. Division of Preventive Medicine, Brigham and Women's Hospital, Boston, Massachusetts 02215, USA

138. Harvard Medical School, Boston, Massachusetts 02115, USA

139. Department of Clinical Chemistry, University of Tampere and Tampere University Hospital, 33520 Tampere, Finland

140. Research Centre of Applied and Preventive Cardiovascular Medicine, University of Turku, 20520 Turku, Finland

141. The Department of Clinical Physiology, Turku University Hospital, 20520 Turku, Finland

142. Division of Research, Kaiser Permanente Northern California, Oakland, California 94612, USA

143. Department of Vascular Medicine, Academic Medical Center, Amsterdam, The Netherlands

144. Centre For Paediatric Epidemiolgy and Biostatistics/MRC Centre of Epidemiology for Child Health, University College of London Institute of Child Health, London, UK

145. Telethon Institute for Child Health Research, West Perth Western Australia 6872, Australia

146. Centre for Child Health Research, The University of Western Australia, Australia

147. PathWest Laboratory of Western Australia, Department of Molecular Genetics, J Block, QEII Medical Centre, Nedlands, Western Australia 6009, Australia

148. School of Pathology and Laboratory Medicine, University of Western Australia, Nedlands, Western Australia 6009,Australia

149. School of Population Health, The University of Western Australia, Nedlands WA 6009, Australia

150. Medizinische Klinik II, Universität zu Lübeck Ratzeburger Allee 160, D-23538 Lübeck, Germany

151. Department of Medicine III, University of Dresden, Medical Faculty Carl Gustav Carus, Fetscherstrasse 74, 01307 Dresden, Germany

152. Department of Public Health and Clinical Nutrition, University of Eastern Finland, Finland

153. Research Unit, Kuopio University Hospital, Kuopio, Finland

154. Institute of Biomedicine/Physiology, University of Eastern Finland, Kuopio Campus, Finland

155. Kuopio Research Institute of Exercise Medicine, Kuopio, Finland

156. Department of Clinical Physiology and Nuclear Medicine, Kuopio University Hospital, Kuopio, Finland

157. Department of Epidemiology and Public Health, University College London, 1-19 Torrington Place, London WC1E 6BT, UK

158. MRC Unit for Lifelong Health & Ageing, London, UK

159. Faculty of Medicine, Institute of Health Sciences, University of Oulu, Oulu, Finland

160. Unit of General Practice, Oulu University Hospital, Oulu, Finland

161. Finnish Diabetes Association, Kirjoniementie 15, 33680, Tampere, Finland

162. Pirkanmaa Hospital District, Tampere, Finland

163. Department of Clinical Sciences, Genetic and Molecular Epidemiology Unit, Skåne University Hospital Malmö, Lund University, Malmö, Sweden

164. Department of Nutrition, Harvard School of Public Health, Boston, MA

165. Department of Public Health & Clinical Medicine, Umeå University,Umeå, Sweden

166. Department of Odontology, Umeå University, Sweden

167. Medical Research Institute, University of Dundee, Ninewells Hospital and Medical School. Dundee, DD1 9SY

168. Clinic of Cardiology, West German Heart Centre, University Hospital of Essen, University Duisburg-Essen, Germany

169. Institute of Human Genetics, University of Bonn, Bonn, Germany

170. Department of Genomics, Life & Brain Center, University of Bonn, Bonn, Germany

171. HUNT Research Centre, Department of Public Health and General Practice, Norwegian University of Science and Technology, 7600 Levanger, Norway

172. Atherosclerosis Research Unit, Department of Medicine, Solna,Karolinska Institutet, Karolinska University Hospital, 171 76 Stockholm, Sweden

173. Cardiovascular Genetics, British Heart Foundation Laboratories, Rayne Building, University College London, London, United Kingdom

174. Department of Pharmacological Sciences, University of Milan, Monzino Cardiology Center, IRCCS, Milan, Italy

175. Unit for Molecular Epidemiology, Helmholtz Zentrum München - German Research Center for Environmental Health, Neuherberg, Germany

176. Institute of Epidemiology II, Helmholtz Zentrum München - German Research Center for Environmental Health, Neuherberg, Germany

177. Hannover Unified Biobank, Hannover Medical School, 30625 Hannover, Germany

178. Department of Internal Medicine II – Cardiology, University of Ulm Medical Center, Ulm, Germany

179. Department of Medicine I, University Hospital Grosshadern, Ludwig-Maximilians-Universität, Munich, Germany

180. Institute of Medical Informatics, Biometry and Epidemiology, Chair of Genetic Epidemiology, Ludwig-Maximilians-Universität, Munich, Germany

181. Division of Endocrinology and Diabetes, Department of Medicine, University Hospital, Ulm, Germany

182. LURIC Study nonprofit LLC, Freiburg, Germany

183. Mannheim Institute of Public Health, Social and Preventive Medicine, Medical Faculty of Mannheim, University of Heidelberg, Mannheim, Germany

184. Synlab Academy, Mannheim, Germany

185. Cardiology Group, Frankfurt-Sachsenhausen, Germany

186. Department of Medicine, University of Kuopio and Kuopio University Hospital, 70210 Kuopio, Finland

187. Department of Epidemiology and Public Health, Faculty of Medicine, Strasbourg, France

188. Department of Clinical Medicine, University of Milano-Bicocca, Monza, Italy

189. Centre for Public Health, Queen's University, Belfast, UK

190. Department of Medical Sciences, Uppsala University, Akademiska sjukhuset, 751 85 Uppsala, Sweden

191. Division of Cardiovascular Epidemiology, Institute of Environmental Medicine, Karolinska Institutet, Stockholm, Sweden

192. Department of Dietetics-Nutrition, Harokopio University, 70 El. Venizelou Str, Athens, Greece

193. 1st Cardiology Department, Onassis Cardiac Surgery Center 356, Sygrou Ave., Athens, Greece

194. Department of Community Medicine, Faculty of Health Sciences, University of Tromsø, Tromsø, Norway

195. Department of Medicine, Stanford University School of Medicine, Stanford, California 94305, USA

196. University of Cambridge Metabolic Research Labs, Institute of Metabolic Science Addenbrooke's Hospital, CB2 OQQ, Cambridge, UK

197. Division of Intramural Research, National Heart, Lung and Blood Institute, Framingham Heart Study, Framingham, Massachusetts 01702, USA

198. Lund University Diabetes Centre, Department of Clinical Sciences, Lund University, 20502 Malmö, Sweden

199. Medical Genetics Institute, Cedars-Sinai Medical Center, Los Angeles, California 90048, USA

200. Channing Laboratory, Department of Medicine, Brigham and Women's Hospital and Harvard Medical School, Boston, Massachusetts 02115, USA

201. Department of Epidemiology and Population Health, Albert Einstein College of Medicine, Bronx, New York 10461, USA

202. Laboratory of Genetics, National Institute on Aging, Baltimore, Maryland 21224, USA

203. Division of Community Health Sciences, St George's, University of London, London, SW17 0RE, UK

204. Center of Medical Systems Biology, Leiden University Medical Center, 2333 ZC Leiden, the Netherlands

205. Oxford National Institute for Health Research Biomedical Research Centre, Churchill Hospital, Old Road Headington, Oxford, OX3 7LJ, UK

206. Genetics of Obesity and Related Metabolic Traits Program,The Charles Bronfman Institute of Personalized Medicine, Child Health and Development Institute, Mount Sinai School of Medicine, New York, NY 10029, USA
