# Supplementary material for: Approaches to detect genetic effects that differ between two strata in genome-wide meta-analyses: Recommendations based on a systematic evaluation
Source: PLoS One. 2017 Jul 27;12(7):e0181038. doi: 10.1371/journal.pone.0181038 (PMC5531538; doi:10.1371/journal.pone.0181038)
Supplement: S1 Table — Stated are the tests that can be applied based on the meta-analyzed stratum-specific genetic effect estimates, β^i, and standard errors, sei (i = 1,2), the respective null hypotheses, test statistics, nomenclature for P-values and the usage. (DOCX) [file pone.0181038.s008.docx]

## Table S1.

| **Name** | **Null hypothesis** | **Test-Statistic** | **P-value** | **Utilization** |
| --- | --- | --- | --- | --- |
| Difference test | $H_{0}:\beta_{1}=\beta_{2}$ | For unrelated individuals:  $Z_{Diff}=\frac{\hat{\beta}_{1}-\hat{\beta}_{2}}{\sqrt{{se}_{1}^{2}+{se}_{2}^{2}}} \sim\left. N\left( 0,1 \right) \right\vert H_{0}$  For related individuals:  $Z_{Diff}=\frac{\hat{\beta}_{1}-\hat{\beta}_{2}}{\sqrt{{se}_{1}^{2}+{se}_{2}^{2}-2\cdot Cov(\hat{\beta}_{1},\hat{\beta}_{2})}} \sim\left. N\left( 0,1 \right) \right\vert H_{0}$ | $P_{Diff}$ | Identify G x S |
| Overall test | $H_{0}:\beta_{Overall}=0$ | $Z_{Overall}=\frac{\hat{\beta}_{Overall}}{{se}_{Overall}}\sim\left. N\left( 0,1 \right) \right\vert H_{0}$, with  $\hat{\beta}_{Overall}=\frac{{\hat{\beta}_{1}}/{{se}_{1}^{2}}+{\hat{\beta}_{2}}/{{se}_{2}^{2}}}{1/{{se}_{1}^{2}}+1/{{se}_{2}^{2}}}$;${se}_{Overall}=\sqrt{\frac{1}{1/{{se}_{1}^{2}}+1/{{se}_{2}^{2}}}}$ | $P_{Overall}$ | Filtering |
| Stratified test | $H_{0}:\beta_{1}=0 \wedge\beta_{2}=0$ | $Z_{1}=\frac{\hat{\beta}_{1}}{{se}_{1}}\sim\left. N(0,1) \right\vert H_{0}$ ; $Z_{2}=\frac{\hat{\beta}_{2}}{{se}_{2}}\sim\left. N(0,1) \right\vert H_{0}$ | $P_{Strat}=2\cdot\min\left( P_{1},P_{2} \right)$  with P_i_ from Z_i_, i=1,2, | Filtering |
| Joint test | $H_{0}:{(\beta}_{1},\beta_{2})=\vec{0}$ | $C_{Joint}=\left( \frac{\hat{\beta}_{1}}{{se}_{1}} \right)^{2}+\left( \frac{\hat{\beta}_{2}}{{se}_{2}} \right)^{2}\sim\left. \chi_{2}^{2} \right\vert H_{0}$ | $P_{Joint}$ | Filtering |
