# Supplementary material for: Approaches to detect genetic effects that differ between two strata in genome-wide meta-analyses: Recommendations based on a systematic evaluation
Source: PLoS One. 2017 Jul 27;12(7):e0181038. doi: 10.1371/journal.pone.0181038 (PMC5531538; doi:10.1371/journal.pone.0181038)
Supplement: S2 Table — The table shows the lead variants identified by the two one-stage approaches [Diff5e-8] and [Overall1e-5→DiffαDiff] that were applied to the sex-stratified GWAMA results for WHRadjBMI (up to 77,000 men and 98,000 women) from the GIANT consortium [12]. Significant sex-difference P-Values (corrected for correlation between strata using r = 0.05 as estimated from the GIANT data on 77,000 men and 98,000 women) are marked in bold. Sex-difference P-Values (uncorrected for correlation) were added to the table for comparison. (DOCX) [file pone.0181038.s009.docx]

## Table S2.

|  |  |  |  |  |  |  |  | **MEN** | | | | **WOMEN** | | | |  |
| --- | --- | --- | --- | --- | --- | --- | --- | --- | --- | --- | --- | --- | --- | --- | --- | --- |
| **Locus** | **Lead-SNP^a^** | **EA** | **[Diff]** | **[Overall**  **🡪Diff]** | **P_Sexdiff_** | **P_Sexdiff_**  **_(uncorrected)_** | **P_Overall_** | **EAF** | **Beta** | **P** | **N** | **EAF** | **Beta** | **P** | **N** | **Type of**  **GxS** |
| *SLC30A10* | 1:217820132 | T | Yes | Yes | **1.2E-16** | **7.0E-16** | 1.2E-23 | 0.72 | 0.0048 | 0.37 | 76,626 | 0.72 | 0.064 | 4.6E-37 | 98,352 | Pure |
| *COBLL1^b^* | 2:165236870 | T |  | Yes | **6.5E-16** | **3.6E-15** | 2.7E-17 | 0.40 | -3.0E-04 | 0.95 | 75,573 | 0.40 | -0.054 | 1.8E-31 | 97,142 | Pure |
|  | 2:165247907 | T | Yes |  | **1.0E-16** | **6.2E-16** | 1.4E-14 | 0.59 | -0.0025 | 0.61 | 76,594 | 0.59 | 0.052 | 2.0E-29 | 98,322 |  |
| *PPARG* | 3:12463882 | C |  | Yes | **2.2E-06** | **4.0E-06** | 2.2E-09 | 0.43 | 0.0042 | 0.41 | 74,653 | 0.43 | 0.037 | 4.2E-14 | 96,473 | Pure |
| *ADAMTS9* | 3:64676186 | A |  | Yes | **4.1E-05** | **6.5E-05** | 4.9E-20 | 0.70 | 0.018 | 9.0E-04 | 75,590 | 0.70 | 0.047 | 5.3E-21 | 97,150 | Quantitative |
| *PLXND1^c^* | 3:130816923 | A |  | Yes | **3.2E-07** | **6.5E-07** | 1.5E-09 | 0.79 | 0.0026 | 0.68 | 74,655 | 0.79 | 0.044 | 7.5E-15 | 96,056 | Pure |
|  | 3:130822305 | T | Yes |  | **5.3E-09** | **1.3E-08** | 1.5E-07 | 0.79 | -0.0073 | 0.33 | 44,701 | 0.79 | 0.048 | 2.4E-14 | 73,628 |  |
| *TNFAIP8* | 5:118757185 | T |  | Yes | **5.2E-06** | **9.0E-06** | 2.6E-08 | 0.29 | -0.0033 | 0.55 | 75,584 | 0.29 | -0.037 | 7.9E-13 | 97,136 | Pure |
| *VEGFA* | 6:43872529 | T | Yes | Yes | **7.1E-12** | **2.5E-11** | 1.6E-22 | 0.47 | 0.010 | 0.049 | 75,704 | 0.47 | 0.060 | 2.4E-31 | 97,269 | Quantitative |
| *NKX3-1* | 8:23659269 | A |  | Yes | **6.5E-07** | **1.3E-06** | 3.8E-07 | 0.77 | -5.0E-04 | 0.94 | 75,598 | 0.77 | 0.038 | 3.4E-12 | 97,157 | Pure |
| *LRRC69* | 8:92217371 | T | Yes |  | **4.1E-08** | 9.1E-08 | 0.95 | 0.67 | -0.026 | 4.4E-05 | 44,792 | 0.68 | 0.019 | 5.4E-04 | 73,744 | Qualitative |
| *ITPR2* | 12:26361436 | T |  | Yes | **1.4E-05** | **2.0E-04** | 2.1E-13 | 0.25 | 0.013 | 0.022 | 74,269 | 0.25 | 0.042 | 4.4E-15 | 95,750 | Quantitative |
| a Format chromosome:base position, build 36; b Different lead SNPs identified by the two approaches (r^2^ = 0.96); c Different lead SNPs identified by the two approaches (r^2^ = 0.97). EA = Effect allele; EAF = Effect allele frequency | | | | | | | | | | | | | | | | |

## 
