# Supplementary material for: Approaches to detect genetic effects that differ between two strata in genome-wide meta-analyses: Recommendations based on a systematic evaluation
Source: PLoS One. 2017 Jul 27;12(7):e0181038. doi: 10.1371/journal.pone.0181038 (PMC5531538; doi:10.1371/journal.pone.0181038)
Supplement: S3 Table — The table shows the lead variants identified by the two-stage approaches [Strat1e-5]→[DiffαDiff] and [Joint1e-5]→[DiffαDiff] that were applied to the sex-stratified GWAMA results for WHRadjBMI (up to 35,000 and 42,000 men; and up to 43,000 and 55,000 women in the two stages respectively; from the GIANT consortium [12]. Significant stage 2 sex-difference P-Values (corrected for correlation between strata using r = 0.03 as estimated from the Stage 2 GIANT data on 42,000 men and 55,000 women) are marked in bold. Stage 2 sex-difference P-Values (uncorrected for correlation) were added to the table for comparison. (DOCX) [file pone.0181038.s010.docx]

## Table S3.

|  |  |  |  |  |  |  |  |  |  | **MEN** | | | | **WOMEN** | | | |
| --- | --- | --- | --- | --- | --- | --- | --- | --- | --- | --- | --- | --- | --- | --- | --- | --- | --- |
| **Nearest**  **Gene** | **LeadSNP^a^** | **EA** | **[Strat]**  **->[Diff]** | **[Joint]**  **->[Diff]** | **Stage** | **P_Joint_** | **P_Strat_** | **P_Sexdiff_** | **P_Sexdiff_**  **_(uncorrected)_** | **EAF** | **Beta** | **P** | **N** | **EAF** | **Beta** | **P** | **N** |
| *SLC30A10* | 1:217820132 | T | Yes | Yes | 1 | 1.7E-17 | 7.4E-18 | NA | NA | 0.72 | 0.0098 | 0.19 | 34,601 | 0.72 | 0.064 | 3.7E-18 | 42,735 |
|  |  |  |  |  | 2 | NA | NA | **3.2E-10** | **5.2E-10** | 0.72 | -6.0E-04 | 0.94 | 42,025 | 0.72 | 0.064 | 1.8E-20 | 55,617 |
| *COBLL1* | 2:165247907 | T | Yes | Yes | 1 | 1.7E-14 | 5.6E-15 | NA | NA | 0.58 | 0.0069 | 0.31 | 34,576 | 0.58 | 0.054 | 2.8E-15 | 42,707 |
|  |  |  |  |  | 2 | NA | NA | **1.2E-11** | **2.2E-11** | 0.60 | -0.013 | 0.070 | 42,019 | 0.60 | 0.051 | 7.3E-16 | 55,615 |
| *ADAMTS9^b^* | 3:64679931 | T |  | Yes | 1 | 4.0E-08 | 2.1E-07 | NA | NA | 0.69 | 0.018 | 0.016 | 34,601 | 0.70 | 0.038 | 1.1E-07 | 42,735 |
|  |  |  |  |  | 2 | NA | NA | **7.4E-04** | **8.6E-04** | 0.70 | 0.018 | 0.019 | 40,995 | 0.70 | 0.053 | 1.8E-14 | 54,422 |
|  | 3:64693298 | C | Yes |  | 1 | 5.1E-08 | 3.3E-08 | NA | NA | 0.28 | -0.0097 | 0.20 | 34,599 | 0.28 | -0.042 | 1.6E-08 | 42,733 |
|  |  |  |  |  | 2 | NA | NA | **3.6E-04** | **4.3E-04** | 0.27 | -0.021 | 0.012 | 38,050 | 0.27 | -0.060 | 1.7E-16 | 52,592 |
| *VEGFA* | 6:43872529 | T | yes | Yes | 1 | 2.9E-13 | 2.2E-13 | NA | NA | 0.47 | 0.012 | 0.11 | 34,594 | 0.47 | 0.057 | 1.1E-13 | 42,727 |
|  |  |  |  |  | 2 | NA | NA | **6.2E-08** | **9.1E-08** | 0.47 | 0.0087 | 0.22 | 41,110 | 0.47 | 0.062 | 2.8E-19 | 54,542 |
| a Format chromosome:base position, build 36; b Different lead SNPs identified by the two filtering tests (r^2^ = 1.0); EA = Effect allele; EAF = Effect allele frequency | | | | | | | | | | | | | | | | | |
