# Supplementary material for: Approaches to detect genetic effects that differ between two strata in genome-wide meta-analyses: Recommendations based on a systematic evaluation
Source: PLoS One. 2017 Jul 27;12(7):e0181038. doi: 10.1371/journal.pone.0181038 (PMC5531538; doi:10.1371/journal.pone.0181038)
Supplement: S4 Table — Instead of applying two stratified linear regression models per study and meta-analyzing stratified genetic estimates, an interaction GWAMA framework involves one interaction model per study, Y=∝ik+βGikGik+βSikSik+βGxSikGikxSik+Eik(j), where S codes strata membership (i.e. S = 0 for stratum 1, S = 1 for stratum 2). Meta-analyzed genetic main effects (β^G) and gene-strata interaction effects (β^GxS) with corresponding standard errors (seG and seGxS) are obtained from study-specific genetic main effects (β^Gik) or gene-strata interaction (β^GxSik) effects, respectively. Stated are the tests that can be applied based on the interaction GWAMA framework, the respective null hypotheses, test statistics, nomenclature for P-values and the usage. (DOCX) [file pone.0181038.s011.docx]

## Table S4.

| **Name** | **Null hypothesis** | **Test-Statistic** | **P-value** | **Utilization** |
| --- | --- | --- | --- | --- |
| Interaction test | $H_{0}:\beta_{GxS}=0$ | $Z_{GxS}=\frac{\hat{\beta}_{GxS}}{{se}_{GxS}} \sim\left. N\left( 0,1 \right) \right\vert H_{0}$ | $P_{GxS}$ | Identify  G x S |
| Overall test | $H_{0}:\beta_{Marginal}=0$ | $Z_{Overall}=\frac{\hat{\beta}_{Marginal}}{{se}_{Marginal}}\sim\left. N\left( 0,1 \right) \right\vert H_{0}$, with  $\hat{\beta}_{Marginal}=\hat{\beta}_{G}+\hat{\beta}_{GxS}\cdot\hat{p}_{S=1}$ and  ${se}_{Marginal}=\sqrt{{se}_{G}^{2}+\frac{\hat{p}_{S=1}\hat{p}_{S=0}}{n}({se}_{GxS}^{2}+\hat{\beta}_{GxS}^{2})+{se}_{GxS}^{2}\cdot\hat{p}_{S=1}^{2}}$, with $p_{S=1}$ being the proportion of individuals with S=1 among all individuals n, and assuming independence of $\beta_{G}$ and $\beta_{GxS}$ | $P_{G}$ | Filtering |
| Stratified test | $H_{0}:\beta_{S=0}=0 \wedge\beta_{S=1}=0$ | $Z_{S=0}=\frac{\hat{\beta}_{S=0}}{{se}_{S=0}}\sim\left. N(0,1) \right\vert H_{0}$ ; $Z_{S=1}=\frac{\hat{\beta}_{S=1}}{{se}_{S=1}}\sim\left. N(0,1) \right\vert H_{0}$,  With: $\hat{\beta}_{S=0}=\hat{\beta}_{G}$ and $\hat{\beta}_{S=1}=\hat{\beta}_{G}+\hat{\beta}_{GxS}$ | $P_{Strat}=2\cdot\min\left( P_{S=0},P_{S=1} \right)$  with P_S=i_ from Z_S=i_, i=0,1 | Filtering |
| Joint test | $H_{0}:{(\beta}_{G},\beta_{GxS})=\vec{0}$ | $C_{Joint}=\left( \begin{matrix} \beta_{G} \\ \beta_{GxS} \end{matrix} \right)^{T}\left( \begin{matrix} {se}_{G}^{2} & Cov(\beta_{G},\beta_{GxS}) \\ Cov(\beta_{G},\beta_{GxS}) & {se}_{GxS}^{2} \end{matrix} \right)^{-1}\left( \begin{matrix} \beta_{G} \\ \beta_{GxS} \end{matrix} \right)\sim\left. \chi_{2}^{2} \right\vert H_{0}$ | $P_{Joint}$ | Filtering |
