# Supplementary figures and images for: Approaches to detect genetic effects that differ between two strata in genome-wide meta-analyses: Recommendations based on a systematic evaluation
Source: PLoS One. 2017 Jul 27;12(7):e0181038. doi: 10.1371/journal.pone.0181038 (PMC5531538; doi:10.1371/journal.pone.0181038)

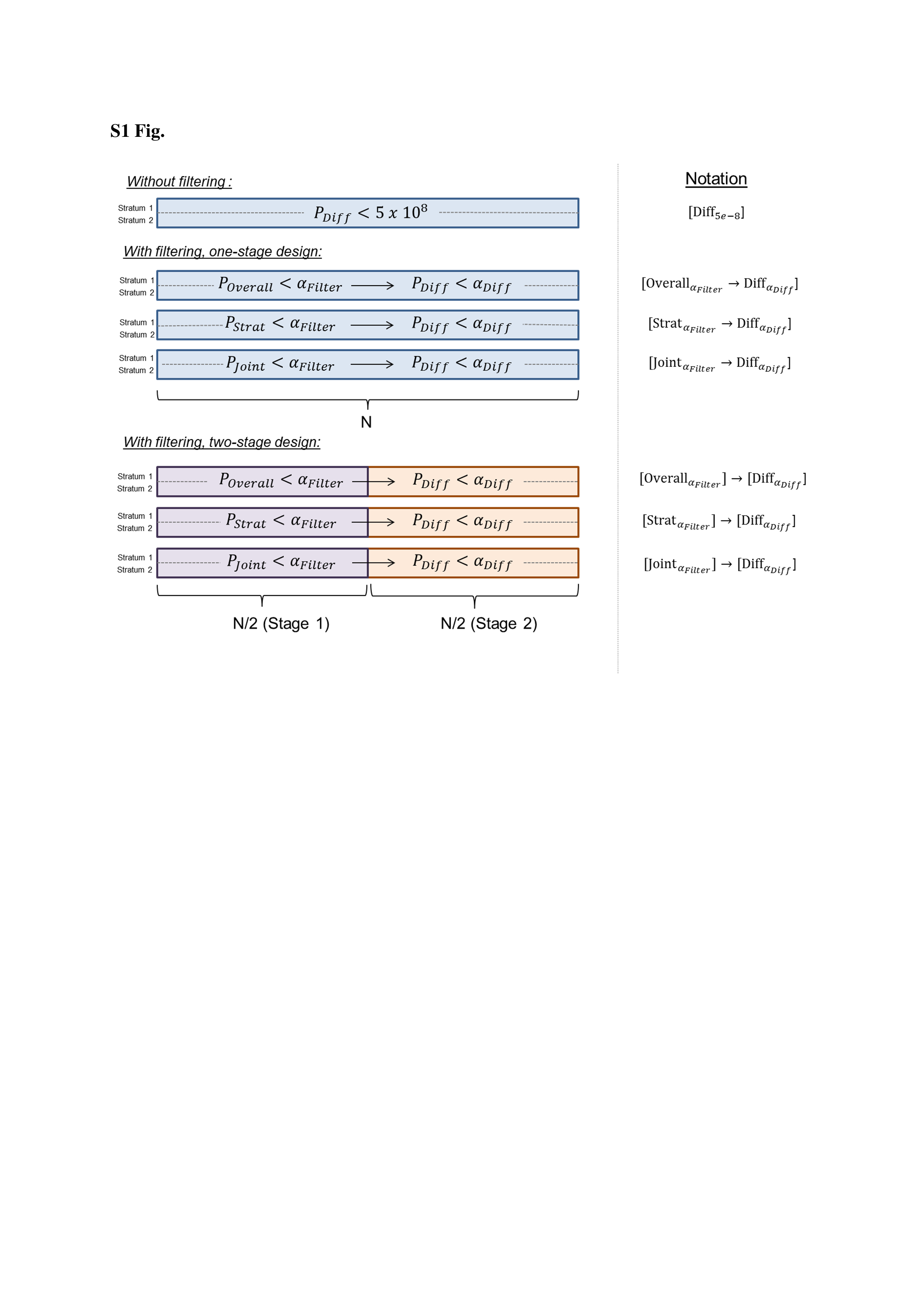

Supplement: S1 Fig — The figure visualizes the approach without filtering as well as the approaches with filtering. The filtering approaches can either be conducted as one-stage or as two-stage approaches. For the one-stage approaches, the filtering and the difference test are applied to one large stratified GWAMA result of total sample size N (blue). For the two-stage approaches, the filtering and the difference test are applied consecutively to two independent stratified GWAMA results of size N/2 (purple and orange). (TIF) [file pone.0181038.s012.tif]

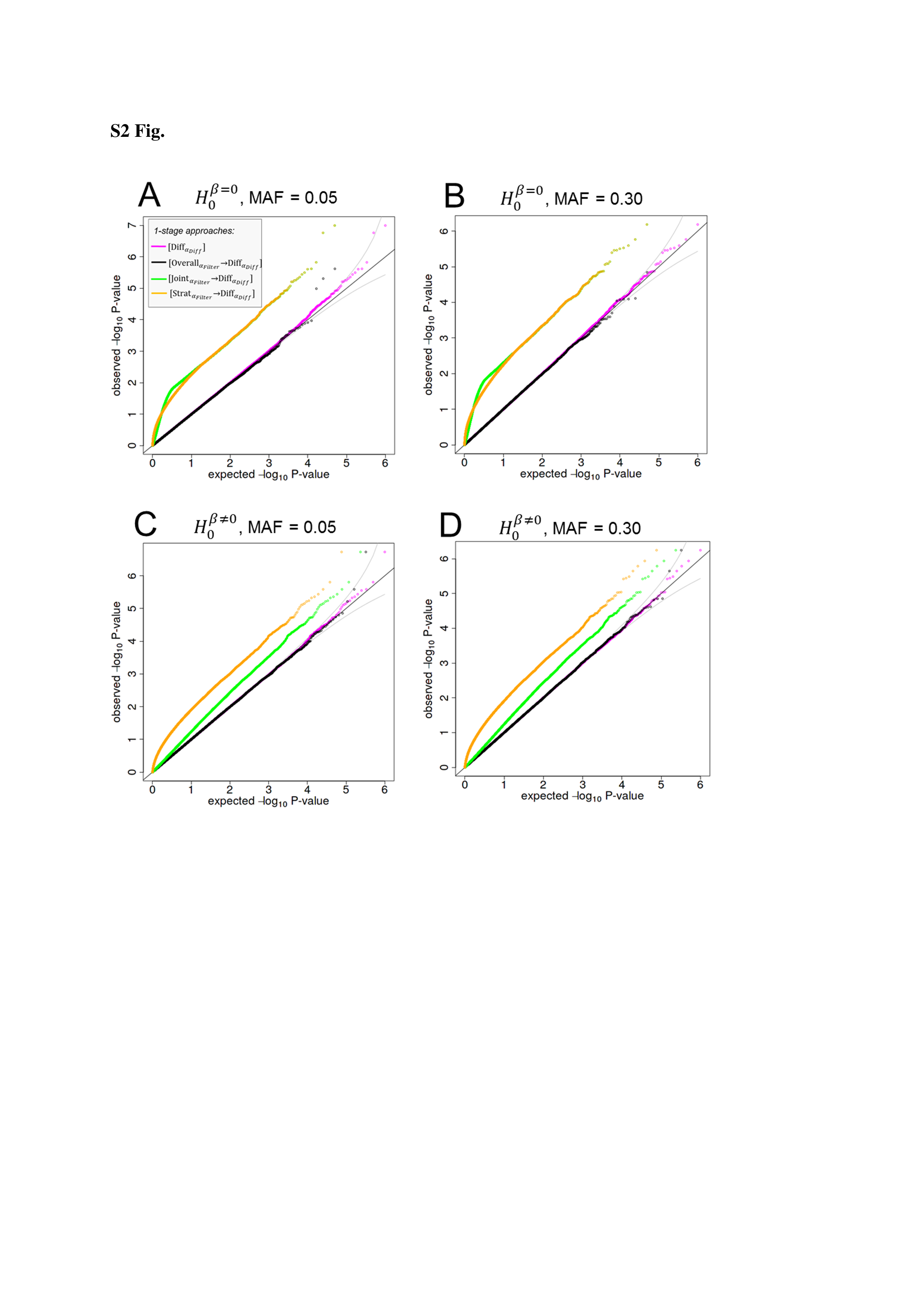

Supplement: S2 Fig — Shown are the difference P-values simulated under the null hypotheses of no GxS given no stratum-specific effects (H0β=0) or of no GxS given identical stratum-specific effects (H0β≠0) for the one-stage approaches [DiffαDiff], [OverallαFilter→DiffαDiff], [StratαFilter→DiffαDiff] and [JointαFilter→DiffαDiff]. The QQ plots are based on simulated phenotypes and simulated genotypes to reflect A) H0β=0 with MAF = 0.05, B) H0β=0 with MAF = 0.30, C) H0β≠0 with MAF = 0.05, and D) H0β≠0 with MAF = 0.30. We here assume αFilter = 0.05 for H0β=0 and αFilter = 10−5 for H0β≠0 and two equally sized (balanced) strata (100,000 individuals in each stratum, f = 1). (TIF) [file pone.0181038.s013.tif]

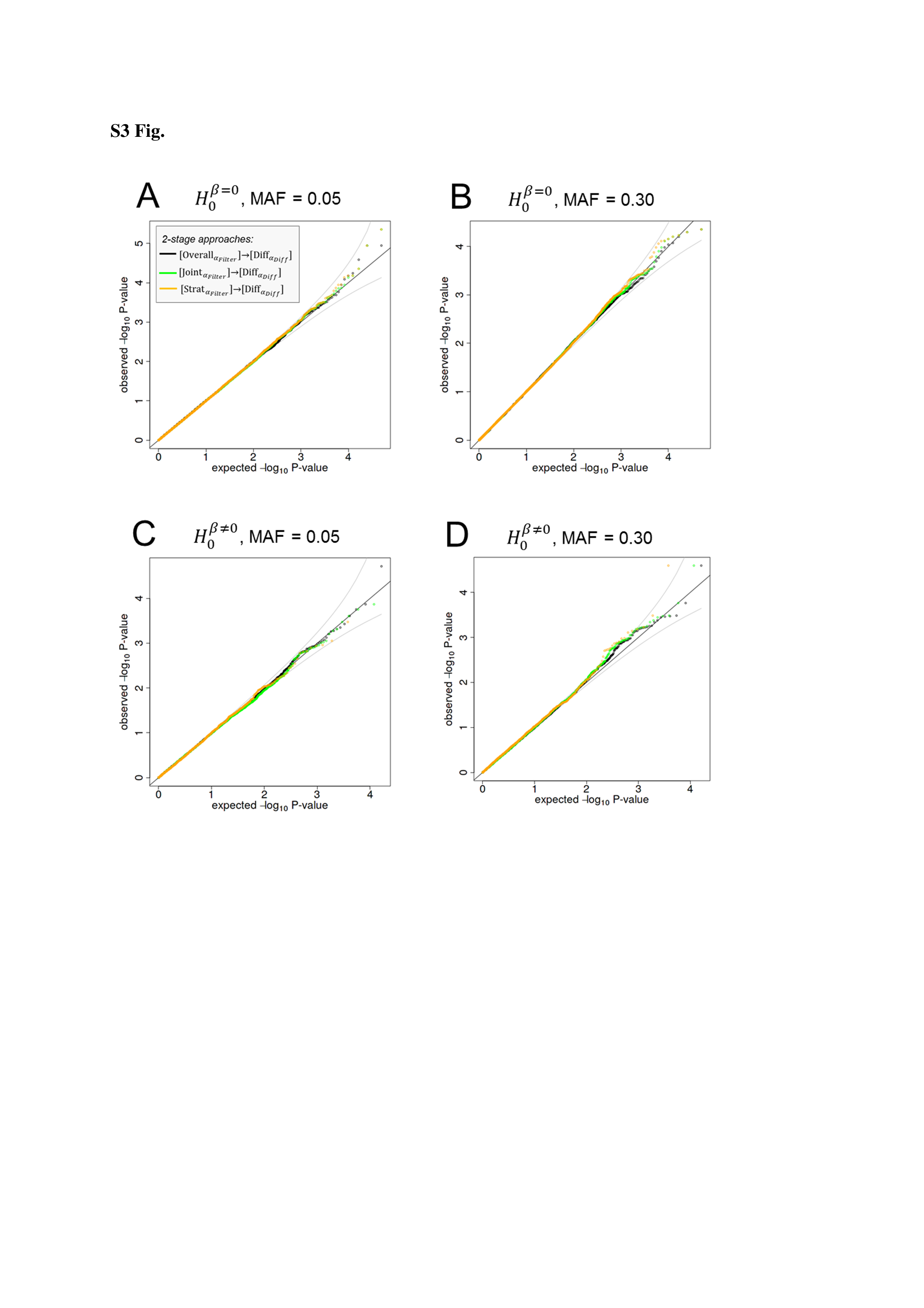

Supplement: S3 Fig — Shown are the simulated difference P-values for the two-stage approaches [OverallαFilter]→[DiffαDiff], [StratαFilter]→[DiffαDiff] and [JointαFilter]→[DiffαDiff]. We here assume two equally sized (balanced) strata (50,000 individuals in each stratum and stage, f = 1). Results are presented for varying MAF and null hypotheses as in S2 Fig. (TIF) [file pone.0181038.s014.tif]

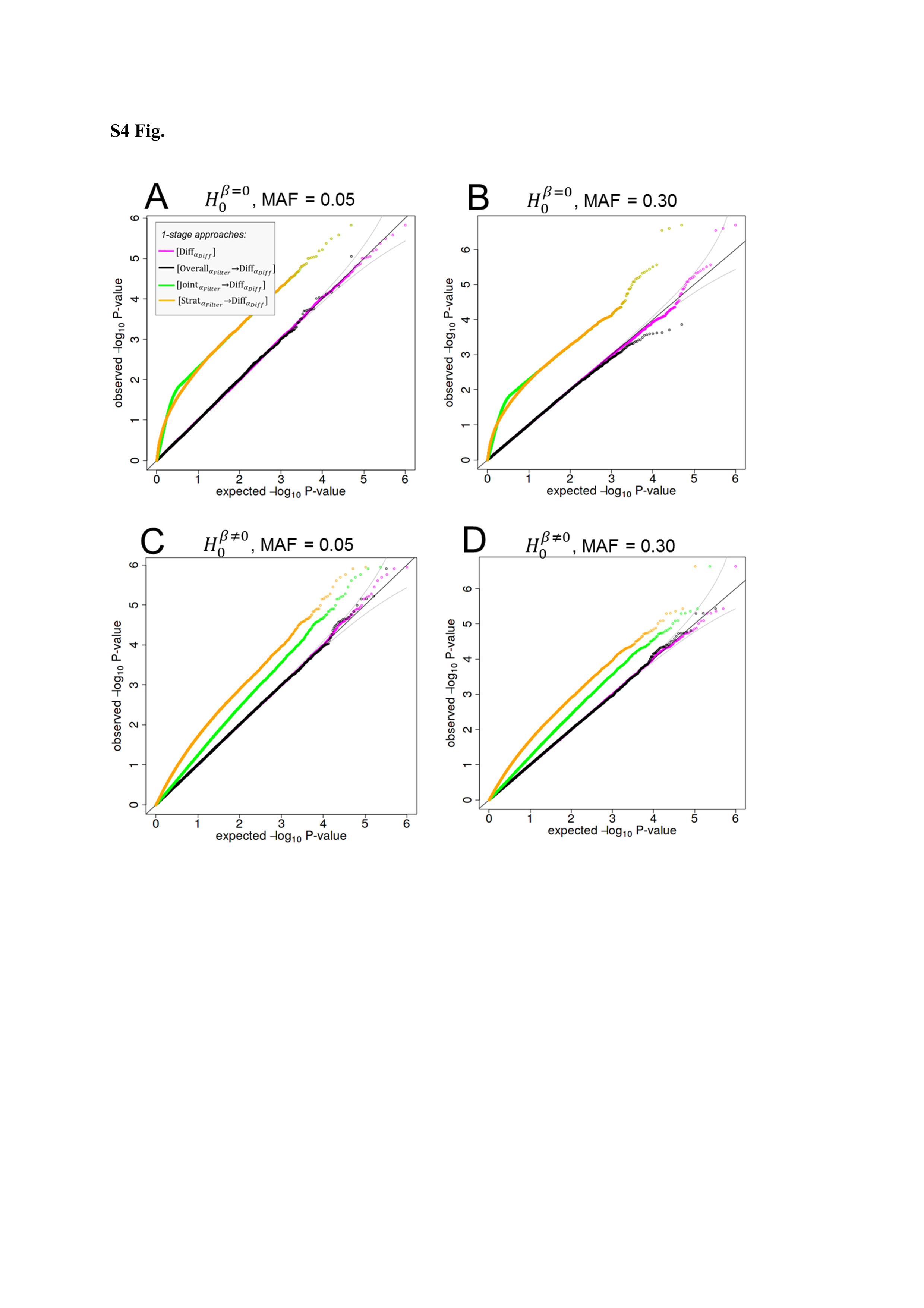

Supplement: S4 Fig — Shown are simulated difference P-values for the one-stage approaches [DiffαDiff], [OverallαFilter→DiffαDiff], [StratαFilter→DiffαDiff] and [JointαFilter→DiffαDiff]. We here assume two unbalanced strata (66,000 and 134,000 individuals in the two strata, respectively, f = 0.33 and f = 3). Results are presented for varying MAF and null hypotheses as in S2 Fig. (TIF) [file pone.0181038.s015.tif]

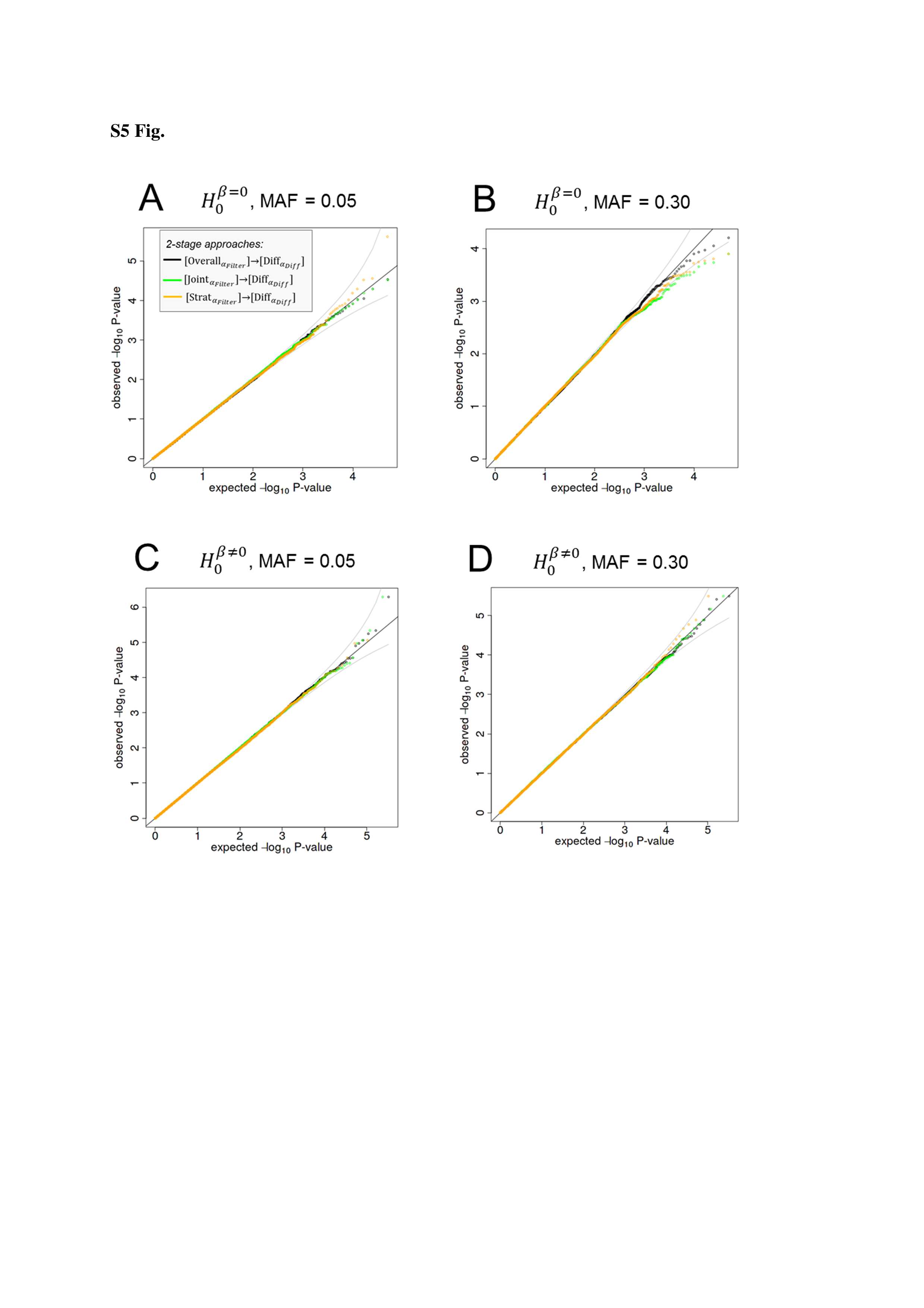

Supplement: S5 Fig — Shown are simulated difference P-values for the two-stage approaches [OverallαFilter]→[DiffαDiff], [StratαFilter]→[DiffαDiff] and [JointαFilter]→[DiffαDiff]. We here assume two unbalanced sized strata (33,000 and 67,000 individuals in the two strata and in each stage, f = 0.33 and f = 3). Results are presented for varying MAF and null hypotheses as in S2 Fig. (TIF) [file pone.0181038.s016.tif]

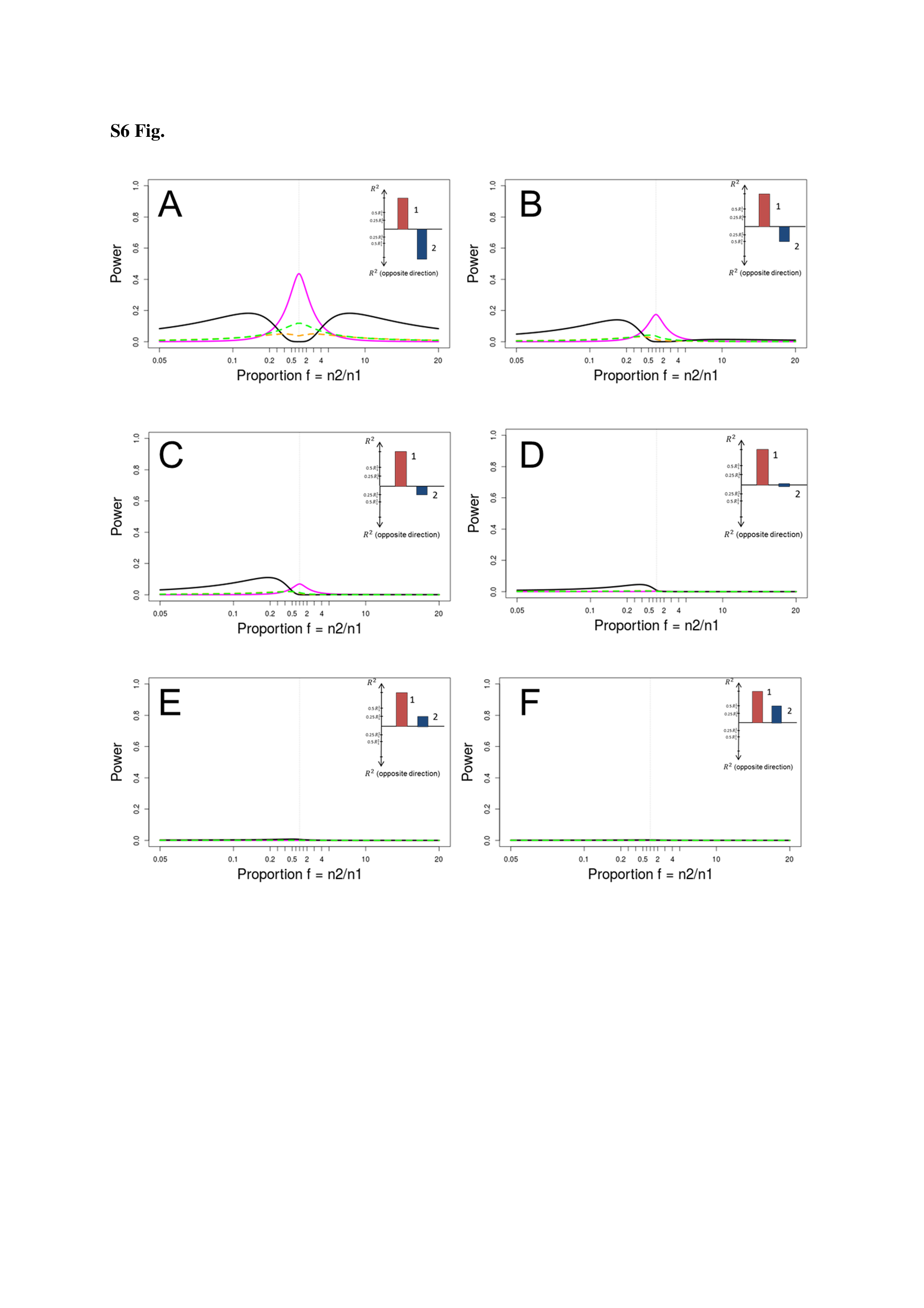

Supplement: S6 Fig — Shown is the power to detect GxS for the same approaches and designs as in Fig 3 (unbalanced strata designs with varying proportion of stratum sample sizes, f = n2/n1, with stratum 1 being the one with the larger effect). Effect size in stratum 1 is fixed to R12=0.014%, as observed for the small WHRadjBMI effect at STAB1. The effect in stratum 2 is fixed to A. 0.014%, into opposite direction (qualitative GxS; same as main Fig 3A), B. R22=0.007%, into opposite direction (qualitative GxS), C. R22=0.003%, into opposite direction (qualitative GxS). D. R22=0% (pure GxS), E. R22=0.003%, into consistent direction (quantitative GxS), and F. R22=0.007%, into consistent direction (quantitative GxS). (TIF) [file pone.0181038.s017.tif]

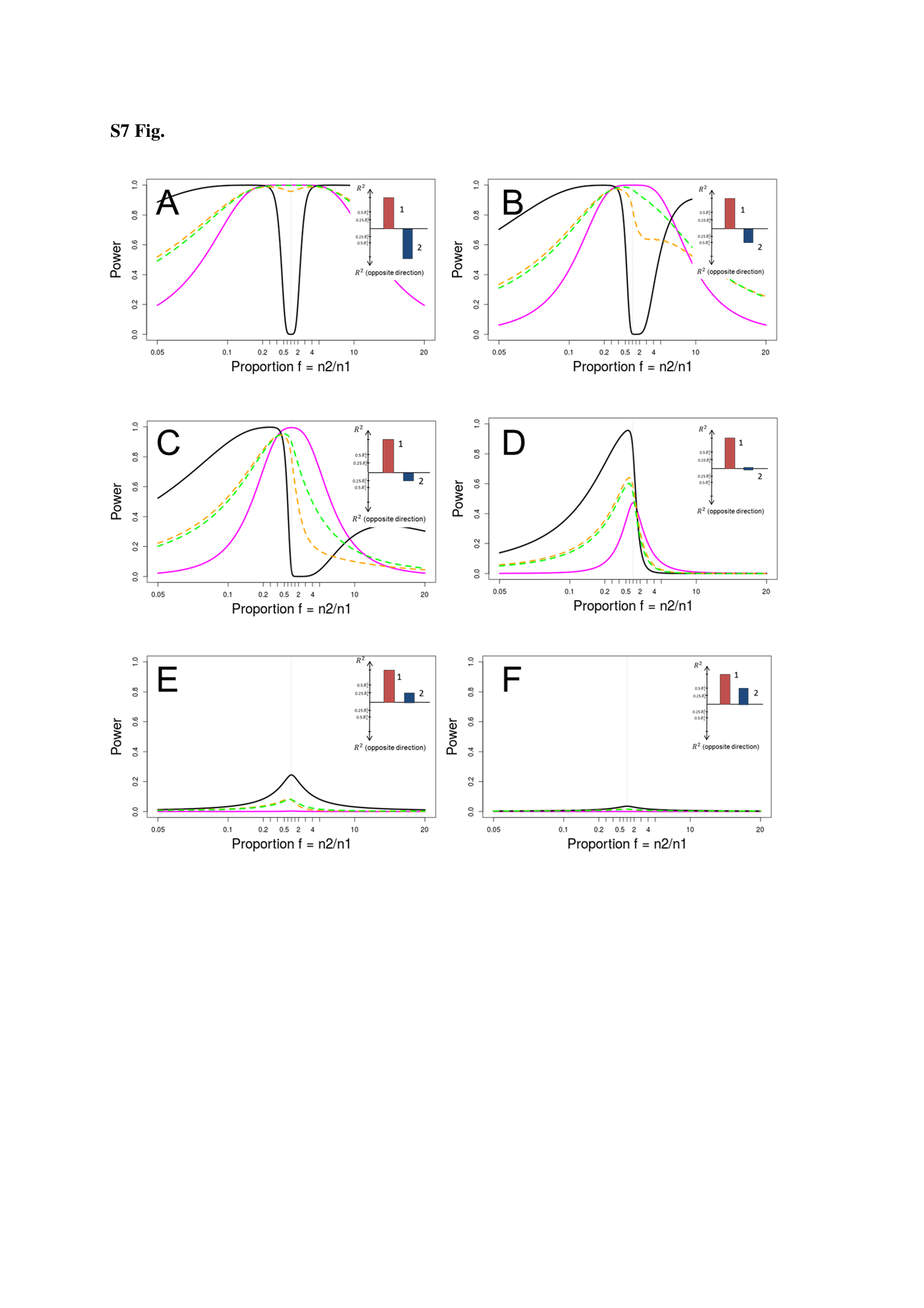

Supplement: S7 Fig — Shown is the power to detect GxS for the same approaches and designs as in Fig 3 (unbalanced strata designs with varying proportion of stratum sample sizes, f = n2/n1, with stratum 1 being the one with the larger effect). Effect size in stratum 1 is fixed to R12=0.058%, as observed for the medium WHRadjBMI effect at PPARG. The effect in stratum 2 is fixed to A. 0.058%, into opposite direction (qualitative GxS), B. R22=0.029%, into opposite direction (qualitative GxS), C. R22=0.014%, into opposite direction (qualitative GxS). D. R22=0% (pure GxS; same as main Fig 3B), E. R22=0.014%, into consistent direction (quantitative GxS), and F. R22=0.029%, into consistent direction (quantitative GxS). (TIF) [file pone.0181038.s018.tif]

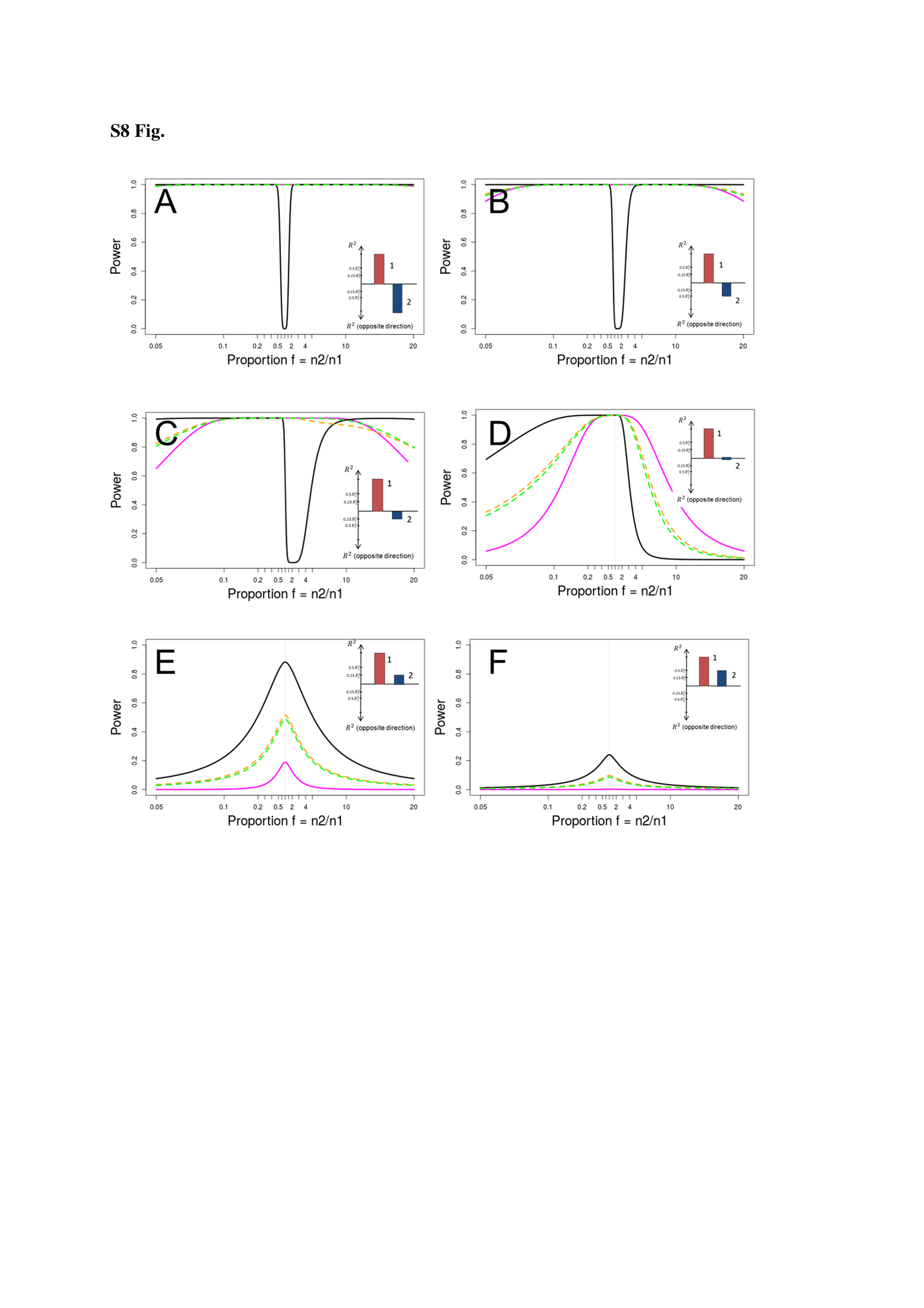

Supplement: S8 Fig — Shown is the power to detect GxS for the same approaches and designs as in Fig 3 (unbalanced strata designs with varying proportion of stratum sample sizes, f = n2/n1, with stratum 1 being the one with the larger effect). Effect size in stratum 1 is fixed to R12=0.0.167%, as observed for the large WHRadjBMI effect at LYPLAL1. The effect in stratum 2 is fixed to A. 0.167%, into opposite direction (qualitative GxS), B. R22=0.084%, into opposite direction (qualitative GxS), C. R22=0.042%, into opposite direction (qualitative GxS). D. R22=0% (pure GxS), E. R22=0.042%, into consistent direction (quantitative GxS, same as main Fig 3C), and F. R22=0.084%, into consistent direction (quantitative GxS). (TIF) [file pone.0181038.s019.tif]

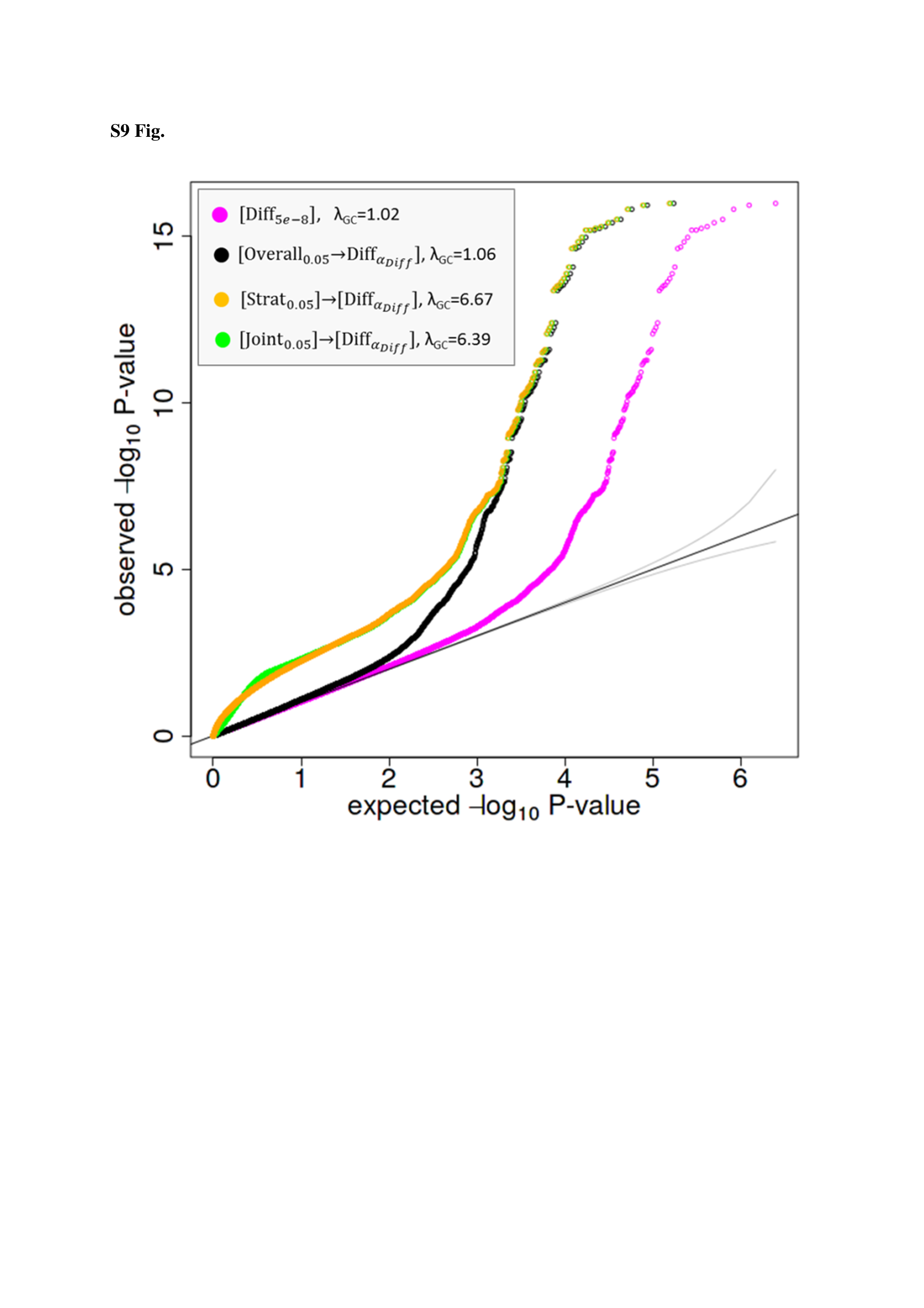

Supplement: S9 Fig — The QQ plot contrasts observed and expected difference P-Values for the considered 1-stage approaches [DiffαDiff], [Overall0.05→DiffαDiff], [Strat0.05→DiffαDiff] and [Joint0.05→DiffαDiff] obtained from an application of approaches to real sex-stratified GWAMA data for WHRadjBMI from the GIANT consortium. (TIF) [file pone.0181038.s020.tif]
